# Time-resolved study of recoil-induced rotation by X-ray pump – X-ray probe spectroscopy†

**DOI:** 10.1039/d1cp05000a

**Published:** 2022-02-24

**Authors:** Ji-Cai Liu, Nina Ignatova, Victor Kimberg, Pavel Krasnov, Alexander Föhlisch, Marc Simon, Faris Gel'mukhanov

**Affiliations:** Department of Mathematics and Physics, North China Electric Power University 102206 Beijing China jicailiu@ncepu.edu.cn; International Research Center of Spectroscopy and Quantum Chemistry - IRC SQC, Siberian Federal University 660041 Krasnoyarsk Russia kimberg@kth.se; Department of Theoretical Chemistry and Biology, KTH Royal Institute of Technology 10691 Stockholm Sweden; Institute for Methods and Instrumentation in Synchrotron Radiation Research FG-ISRR Helmholtz-Zentrum Berlin für Materialien und Energie Albert-Einstein-Strasse 15 12489 Berlin Germany; Institut für Physik und Astronomie, Universität Potsdam, Karl-Liebknecht-Strasse 24-25 14476 Potsdam Germany; Sorbonne Université, CNRS, Laboratoire de Chimie Physique-Matiére et Rayonnement, LCPMR F-75005 Paris France

## Abstract

Modern stationary X-ray spectroscopy is unable to resolve rotational structure. In the present paper, we propose to use time-resolved two color X-ray pump–probe spectroscopy with picosecond resolution for real-time monitoring of the rotational dynamics induced by the recoil effect. The proposed technique consists of two steps. The first short pump X-ray pulse ionizes the valence electron, which transfers angular momentum to the molecule. The second time-delayed short probe X-ray pulse resonantly excites a 1s electron to the created valence hole. Due to the recoil-induced angular momentum the molecule rotates and changes the orientation of transition dipole moment of core-excitation with respect to the transition dipole moment of the valence ionization, which results in a temporal modulation of the probe X-ray absorption as a function of the delay time between the pulses. We developed an accurate theory of the X-ray pump–probe spectroscopy of the recoil-induced rotation and study how the energy of the photoelectron and thermal dephasing affect the structure of the time-dependent X-ray absorption using the CO molecule as a case-study. We also discuss the feasibility of experimental observation of our theoretical findings, opening new perspectives in studies of molecular rotational dynamics.

## Abstract 2

We propose two color X-ray pump–probe spectroscopy, which opens new perspectives in studies of molecular rotational dynamics induces by the recoil effect in real-time. The feasibility of experimental observation is also discussed.

## Graphical abstract

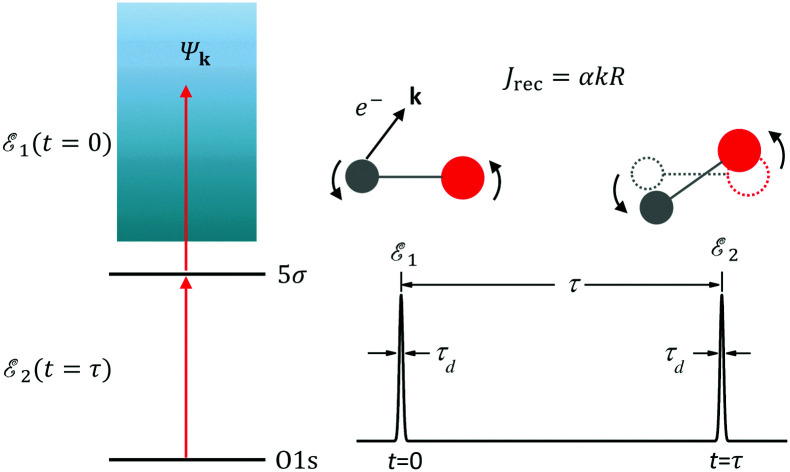

## 1 Introduction

There has been a significant research interest in controlling and imaging molecular translational and rotational dynamics. Control over motion of a single molecule using the scanning tunneling microscope (STM) tip is the key to operation of surface-adsorbed molecular machines.^1,2^ Time-resolved pump–probe measurements allow the fast laser-induced unidirectional molecular rotation to be studied using the “optical centrifuge” technique^3,4^ and real-time monitoring of laser-induced molecular alignment and orientation.^5,6^ The study of rotational structure is a conventional subject of stationary microwave spectroscopy,^7^ which requires neither electronic nor vibrational excitation. Moreover, infrared (IR), optical, and ultraviolet (UV) spectroscopies^7^ are also broadly used for rotational studies, thanks to the long lifetime of the valence electronic and vibrational excited states and to the high spectral resolution in that spectral range. In principle, the rotational structure can be also resolved in resonant inelastic X-ray scattering (RIXS) or resonant Auger scattering (RAS),^8^ as the rotational frequencies are larger than the small lifetime broadening of the final valence-excited state of the scattering process. Unfortunately, the limited resolution of conventional X-ray spectrometers does not allow the rotational structure to be resolved. However, there are two X-ray spectroscopic techniques which allow molecular rotation to be probed. The first one is rotationally resolved optical fluorescence from a molecule ionized by X rays, which allows recoil-induced molecular rotation to be probed.^9^ Secondly, the recoil-induced rotation of a molecule triggered by ejection of an electron was recently probed using Auger spectroscopy,^11^ where the Auger decay was inherently delayed with respect to the photoionization by the lifetime of the core-ionized state. The bottleneck of stationary X-ray spectroscopy for studies of rotational dynamics lies in the very small rotational frequency when compared to spectral resolution in the X-ray range. However, the small rotational frequencies (or large period of molecular rotation) becomes an advantage for time-resolved pump–probe spectroscopy as it does not require large temporal resolution.

In the present paper we theoretically study the molecular rotation induced by X-ray photoionization using two-color all X-ray pump–probe spectroscopy. Upon ejection of a fast photoelectron, a molecule gains a recoil momentum, which not only kicks the molecule in the opposite direction, but may also induce its rotation around the center of mass. Although the photoelectron recoil effect does not create unidirectional rotation, we show that the angular recoil induces a recurrent modulation of the probe X-ray absorption signal. We study in detail the effect of initial rotational temperature and energy of the ejected photoelectron on the temporal profile of the probe signal.

The paper is organized as follows. In Section 2, we present the physical model of the studied two colour all X-ray pump–probe (XX–PP) process taking into account the nuclear degrees of freedom of a molecule using a time-dependent wave packet technique. The general theory of the XX–PP process in Section 2.1 is followed by the theory of the studied pump–probe process for non-overlapping short pump and probe pulses in Section 2.2. The effect of thermal population of initial rotation levels is discussed in Section 2.2.2. We highlight in Section 2.3 the mechanism of the recoil-induced rotational dynamics. The role of vibrations is elucidated in Section 2.3.3. Numerical simulations for the showcase CO molecule and the discussion of the role of the photoelectron energy and thermal dephasing on the recoil-induced recurrent structures are discussed in Section 3. Our findings are summarized in Section 4. To make the presentation more fluent we moved heavy derivations to the ESI.†

## 2 Theoretical model

**Fig. 1:**
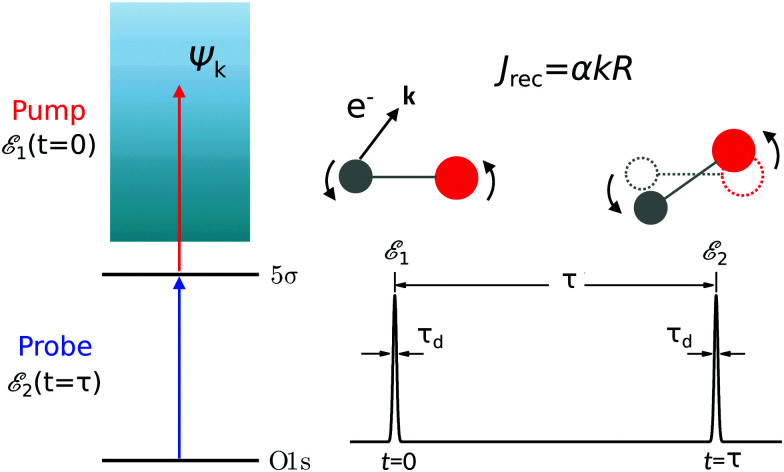
Scheme of the electronic transitions (left panel) in the studied pump–probe process with two time delayed X-ray pulses (right panel). The pump X-ray pulse 
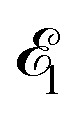
 (red arrow) at the instant *t* = 0 ionizes the electron from the HOMO 5σ to the continuum state *ψ*_**k**_. The ejected photoelectron with the momentum **k** transfers to the molecule recoil-induced angular momentum *J*_REC_ = *α***R** × **k** (*J*_REC_ = *J*_rec_sin *θ*). Then, the time delayed probe X-ray pulse 
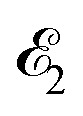
 (blue arrow) at time *t* = *τ* promotes the core electron of the oxygen atom O1s to the previously created valence hole in HOMO *ψ*_5σ_ and probes the recoil-induced rotational dynamics.

The time-resolved two-color X-ray pump–probe process studied here is shown in Fig. 1. We consider the interaction of a molecule with X-ray pump 
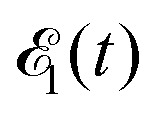
 and time-delayed X-ray probe 
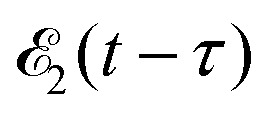
 pulses1
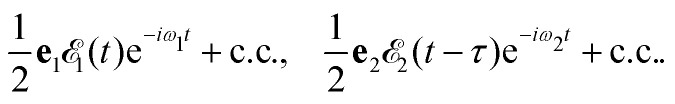
Here **e**_*i*_ and *ω*_*i*_ are the polarization vector and frequency of the X-ray field 
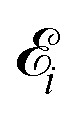
 of the pulse with duration *τ*_d_. We assume that both linearly polarized fields have moderate intensity. To develop a theory of the proposed XX–PP process let us consider a case study of the CO molecule, which has an electronic ground state *Ψ*_0_ = |X^1^Σ^+^〉 = |1σ^2^2σ^2^3σ^2^4σ^2^1π^4^5σ^2^〉. The pump X-ray pulse 
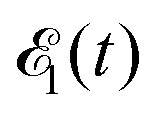
 ionizes an electron from the highest occupied molecular orbital (HOMO) 5σ. The frequency *ω*_10_ of the transition 5σ → *ψ*_**k**_ is the sum of ionization potential *I*_5σ_ of 5σ electron and the kinetic energy of the photoelectron *ε*2
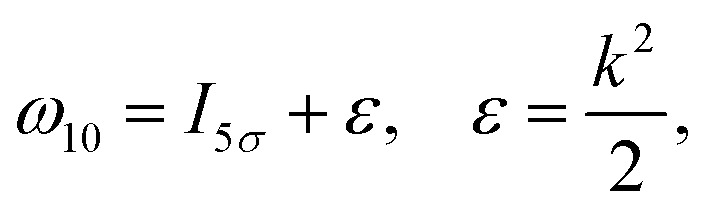
where *ψ*_**k**_ is the wave function of photoelectron with momentum **k**. We neglect the small shift of *I*_5σ_ due to the energy of the initial rotational state *ε*_*J*_0__. The time-delayed probe X-ray pulse 
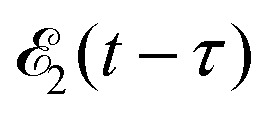
 promotes an 1s core electron to the created valence hole in the 5σ molecular orbital (Fig. 1). To be specific, let us consider here the O1s → 5σ core-excitation with the resonant frequency *ω*_21_ = 528.1 eV.^12^ Thus we will study the following pump–probe process
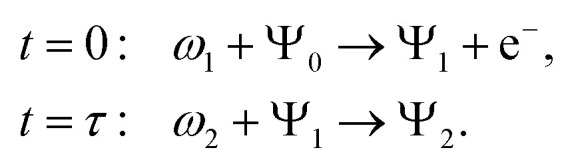
We mark the ground *Ψ*_0_ = |1σ^2^2σ^2^3σ^2^4σ^2^1π^4^5σ^2^〉, valence-ionized *Ψ*_1_ = |5σ^−1^〉 and final core-ionized *Ψ*_2_ = |O1s^−1^〉 states by indexes *i* = 0, 1, and 2, respectively. It is worthwhile to note that the dominating X-ray absorption transition O1s → 2π with resonant energy of 533.4 eV^12^ from the ground state of CO does not mask the X-ray transition O1s → 5σ with resonant energy of 528.1 eV^12^ in the cation CO^+^(5σ^−1^), having smaller transition energy.^13^ It is worth noting, that the pump X-ray pulse can ionize any valence electron (*e.g.* 1π, 4σ, *etc.*) and not only 5σ. However, since the probe X-ray pulse is tuned in resonance with the O1s → 5σ transition it selects only the targeted pump–probe channel (5σ → *ψ*_**k**_; O1s → 5σ).

Instead of the process presented in Fig. 1, where ionization of the highest occupied molecular orbital (HOMO) 5σ is considered, one can also consider schemes with the valence hole in the lower molecular orbitals (1π, 4σ, *etc.*). The only difference with the presented scheme is the much shorter lifetime 1/2*γ* of these intermediate states *Ψ*_1_ in comparison with |*Ψ*_1_〉 = |5σ^−1^〉. This is because the |5σ^−1^〉 state is the ground electronic state of the CO^+^ cation.

Absorption of the pump X-ray photon induces ejection of the fast photoelectron from the HOMO of the CO

3 *ψ*_5σ_ = *ψ*_O,5σ_ + *ψ*_C,5σ_,

which is a coherent superposition of the the atomic orbitals of the oxygen *ψ*_O,5σ_ and carbon *ψ*_C,5σ_ atoms (see eqn (S10) of ESI†). Consequently, the ionization cross-section is the sum of three terms (see Section II of ESI†)

4 *σ*_O_ + *σ*_C_ + *σ*_int_,

where *σ*_O_ and *σ*_C_ are the partial ionization cross-sections of the oxygen and carbon atoms, respectively, and *σ*_int_ is a contribution caused by the interference of these ionization channels. This in nothing else than the Cohen-Fano interference.^14–16^ The interference term for randomly oriented molecules is negligibly small^14–16^ (see Section III of ESI†)5
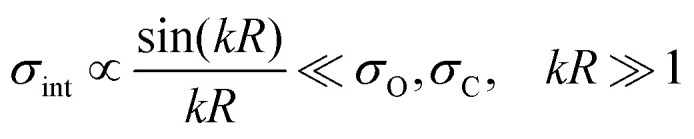
in the energy region studied in the present article. Here **R** is the radius vector between carbon and oxygen atoms. Since the ionization cross-section is simply the sum of the ionization cross-section from the oxygen and carbon atoms *σ*_O_ + *σ*_C_, we will analyze below the partial contribution of the *n*-th atom. All vectors **e**_1_, **e**_2_, **k**, and **R** are defined in the laboratory frame.

Ejection of a fast photoelectron from the *n*-th atom is accompanied by recoil which results in a recoil-induced molecular rotation characterized by the maximal value

6 *J*^(*n*)^_rec_ = *α*_*n*_*kR*, *n* = C, O

of the recoil angular momentum **J**^(*n*)^_REC_ = *α*_*n*_**R** × **k** (*J*^(*n*)^_REC_ = *J*^(*n*)^_rec_sin *Θ*) and the angle *Θ* = ∠(**k**,**R**) between **k** and **R**.^11,16^ Here *α*_C_ = *m*_O_/(*m*_C_ + *m*_O_) = 0.571, *α*_O_ = *m*_C_/(*m*_C_ + *m*_O_) = 0.429, *m*_C_ and *m*_O_ are masses of the carbon and oxygen atoms, respectively. Below we will see that the intensity of the probe absorption, which is the main subject of our study, does not depend on the angle *Θ*. This is because the absorption of the probe X-ray pulse is formed by contributions from molecules with all orientations of **R** for all directions **k** of ejection of the photoelectron. Transfer of the recoil angular momentum creates a rotational wave packet of the molecule, whose evolution can be effectively studied by the second delayed probe pulse in the framework of XX–PP spectroscopy, as discussed in details below. Here and below the contribution from the momentum of the incident X-ray photon is not included due to its negligibly small value in the studied energy range.^16^

It is useful to outline the structure of the derivations for the absorption cross-section of the short time-delayed X-ray pulse in diatomics, given in the following Sections:

(1) In Section 2.1 we solve the amplitude equations for the studied process (Fig. 1) and obtain a general expression for the time-delayed absorption cross-section *σ*_**k**_(*τ*,*t*) of the probe X-ray pulse by a molecule in its initial rovibrational state |*J*_0_*M*_0_,0〉 with the rotational and vibrational degrees of freedom and fixed momentum of the photoelectron ionized by the pump X-ray pulse.

(2) In the beginning of Section 2.2 we compute *σ*_**k**_(*τ*,*t*) for the non-overlapping pump and probe X-ray pulses.

(3) In Section 2.2.1 we consider the important case of short pump and probe pulses and compute the probe absorption *σ*(*τ*). In this case the rovibronic wave packet evolves only between the pulses which results in a drastic simplification of the expression for the probe signal. Moreover, we show that the dynamics of the vibrational wave packet does not affect the probe absorption (see also Section 2.3.3).

(4) At the next step (Section 2.2.2) we take into account thermal population of the initial rotational levels.

(5) In Section 2.3 we analyze dynamics of the recoil-induced rotation using photoelectron local phase factor as a reason for the recoil effect. Here we focus on the specific case of CO molecule, considering the structure of transition dipole moments for the pump and probe pulses.

(6) In Section 2.3.1 we describe the connection between the recoil-induced angular momentum and the momentum of the photoelectron.

(7) The final expression for the probe X-ray signal derived in Section 2.3.2 shows the interference in time domain between the recoil-induced rotational levels, which is manifested as the rotational revivals discussed in Section 3.

### 2.1 General theory

The interaction of the pump 
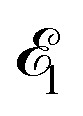
 and probe 
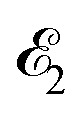
 X-ray pulses (1) with the molecule mixes the ground, intermediate valence-ionized, and the final core-ionized states. Therefore, the total molecular wave function in the interaction picture reads
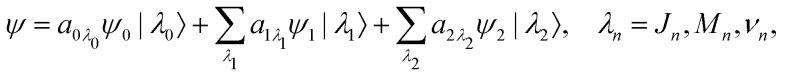
where *ψ*_*n*_ and |*λ*_*n*_〉 = |*J*_*n*_*M*_*n*_,*ν*_*n*_〉 are the electronic and nuclear wave functions, respectively. The nuclear wave function, in turn, is the product of rotational |*J*_*n*_*M*_*n*_〉 = *Y*_*J*_*n*_*M*_*n*__ and vibrational |*ν*_*n*_〉 wave functions. In the present study we assume that only the lowest vibrational level is populated in the ground state *ν*_0_ = 0. To make the presentation more comprehensible|*λ*_0_〉 = |*J*_0_*M*_0_,0〉we start from one initial rotational state |*J*_0_*M*_0_〉 = *Y*_*J*_0_*M*_0__. The general case of thermal population of many initial rotational states will be considered below in Section 2.2.2. Since our approach is limited to weak probe and pump pulses, we neglect depopulation of the ground state assuming *a*_0*λ*_0__ = 1. The amplitudes of the intermediate *a*_1*λ*_1__ and final *a*_2*λ*_2__ states satisfy the following system of equations in the rotating wave approximation (in atomic units)7
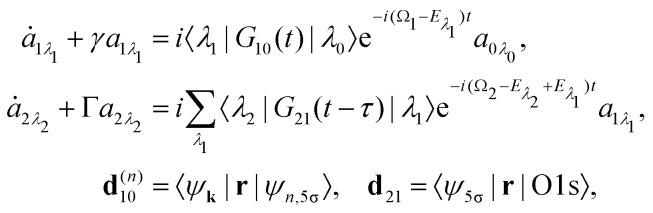
where the nuclear energy *E*_*λ*_*i*__ of the *i*-th electronic state is the sum of rotational and vibrational energies, 
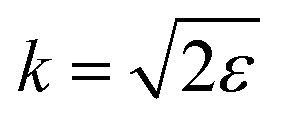
, *Ω*_1_ = *ω*_1_–*ω*_10_ and *Ω*_2_ = *ω*_2_–*ω*_21_ are the detuning of the frequencies of pump and probe fields relative to the frequencies of pump (*ω*_10_) and probe (*ω*_21_) electronic transitions, *ω*_21_ is the spacing between the bottoms of the potential energy curves of the final and intermediate electronic states; *γ* and *Γ* are the lifetime broadening of the valence- and core-ionized electronic states, *ψ*_O,5σ_ and *ψ*_C,5σ_ are defined in eqn (3). The strengths of the interaction with the pump and probe pulses are defined by the Rabi frequencies 
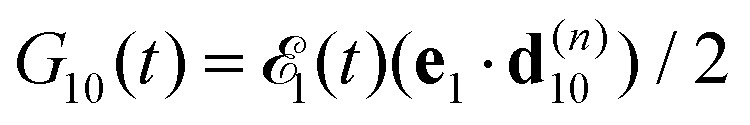
 and 
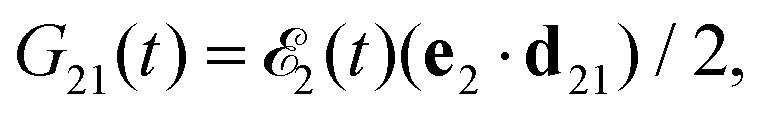
 respectively.

Our main interest is the absorption probability of the second probe X-ray pulse on the transition 1 → 28
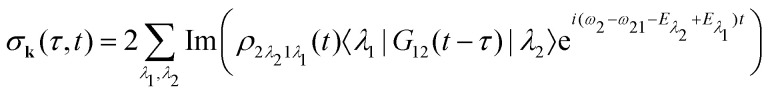
which is defined by the off-diagonal element of the density matrix 
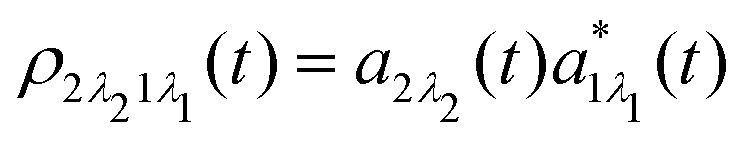
. Taking into account the solution of the amplitude eqn (7)9
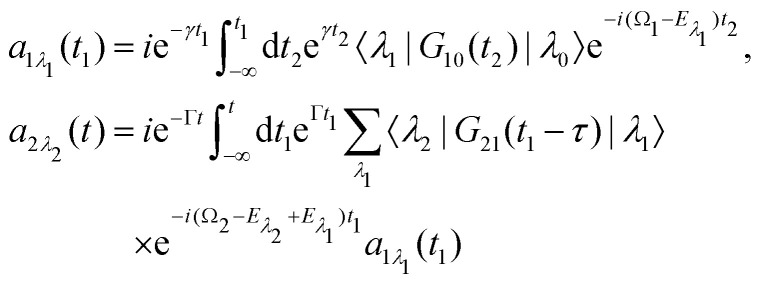
and eqn (S28) of ESI,† the probability (8) can be written in the following compact form10
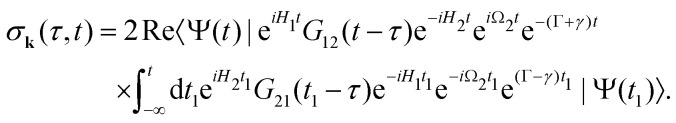
We introduced here the nuclear Hamiltonians *H*_1_ and *H*_2_ of the intermediate and final states, respectively. The nuclear Hamiltonian of the *i*-th electronic state

11 *H*_*i*_ = *h*^vib^_*i*_ + *h*^rot^_*i*_

is the sum of the vibrational *h*^vib^_*i*_ and rotational *h*^rot^_*i*_ Hamiltonians with the eigenvalues *ε*_*ν*_*i*__ and *ε*_*J*_*i*__, respectively. The nuclear wave packet created by the pump X-ray pulse reads12
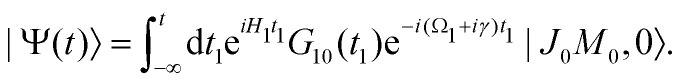


### 2.2 Non-overlapping pump and probe X-ray pulses

The general eqn (10) for absorption cross section of the probe signal can be solved numerically. However, it is instructive to derive an analytical expression by applying some reasonable approximations. For this, let us consider non-overlapping X-ray pulses, when the delay time *τ* exceeds significantly the pulse duration *τ*_d_ (*τ* ≫ *τ*_d_). In that case, the absorption of the probe X-ray pulse happens at times *t* ≈ *τ* ≫ *τ*_d_, where |*Ψ*(*t*)〉 = |*Ψ*(∞)〉. Since the lifetime broadening of valence ionized states is rather small in general, the product *γτ*_d_ ≪ 1 and thus we can neglect exp(*γt*_1_) in expression (12) for |*Ψ*(∞)〉13
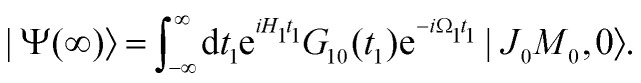
The XX–PP probability should be integrated over the time domain covering the probe X-ray pulse
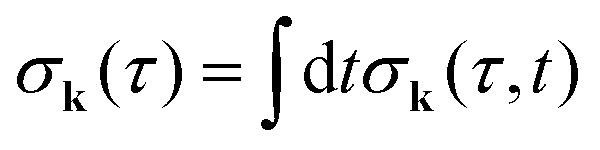
Making use of the replacement *t* → *t* + *τ*, *t*_1_ → *t*_1_ + *τ* we obtain14
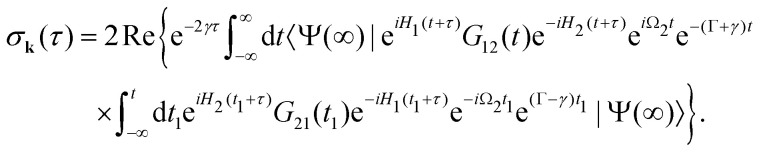


#### 2.2.1 Short pump and probe pulses

In this article we study the interaction of molecules with short pump and probe pulse so that the nuclear wave packet does not have time to change its shape during the exposing of the X-ray pulses. More precisely, we assume that the pulse duration *τ*_d_ is shorter than the characteristic time 1/*E*_*λ*_*n*__ of the evolution of the rovibrational wave packet in the intermediate and final states

15 *τ*_d_*E*_*λ*_*i*__ ≪ 1, *i* = 1, 2.

With this assumption one can replace e^*iH*_1_*t*^*G*_10_(*t*) by *G*_10_(*t*) in eqn (13), and replace e^*iH*_1_*t*^*G*_12_(*t*)*e*^−*iH*_2_*t*^ by *G*_12_(*t*) in eqn (14), then the equation for probability (14) takes the form16
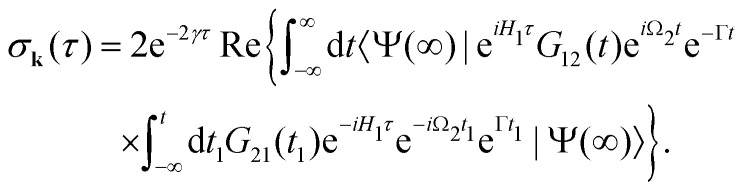
with 
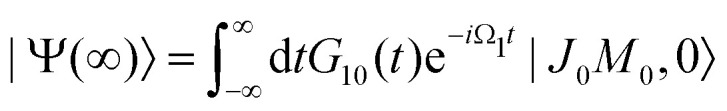
. Since the photoelectron is not detected in the studied process this probability should be integrated over the final states of the photoelectron (momentum *k* and spin)
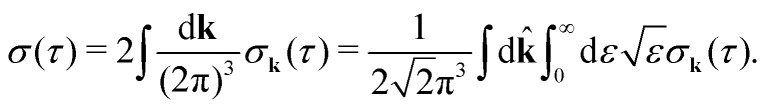
Here **k̂** = **k**/*k* is the unit vector along **k**. To be specific let us consider X-ray pulses having a Gaussian temporal envelope17
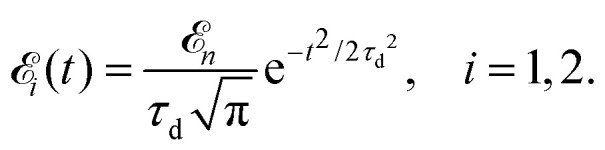
Taking into account eqn (S6) of ESI,†*Ω*_1_ = *ω*_1_ − *I*_5σ_ − *ε* (see eqn (2)) and 
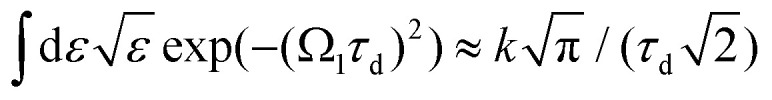
 we obtain18
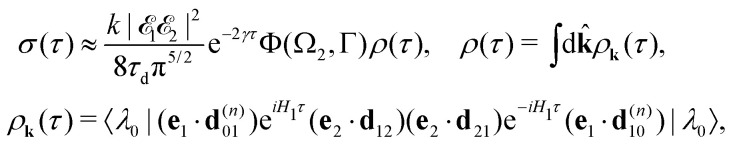
where19
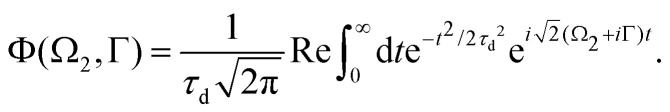
is the Voigt function and 
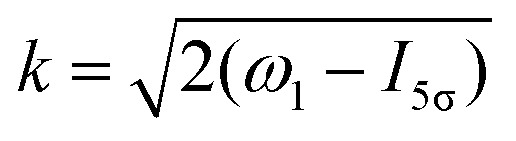
. The rovibrational states of the nuclear Hamiltonian (11) of the intermediate state are the product of rotational *Y*_*J*_1_*M*_1__(**R̂**) and vibrational |*ν*_1_〉 states
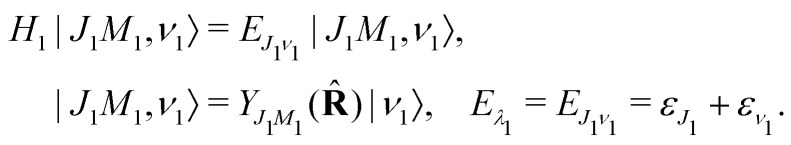
Therefore, it follows that the delay time dependence is defined by the beating between interfering rovibrational levels of the pumped electronic state20
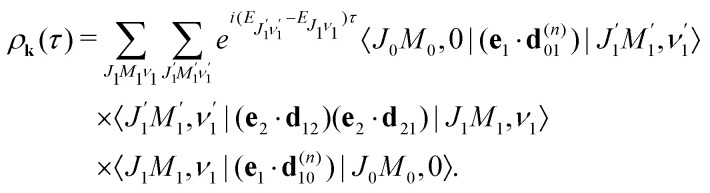
Now we are in stage to discuss the role of the vibrational and rotational degrees of freedom on the time dependence of the probe signal.

The transition dipole moment **d**_21_ of the probe transition O1s → 5σ being an almost atomic matrix element (see eqn (7) and Fig. 1) only weakly depends on the elongation of the bond *R*. Due to this we neglect the change of the vibrational quantum number in the matrix element21
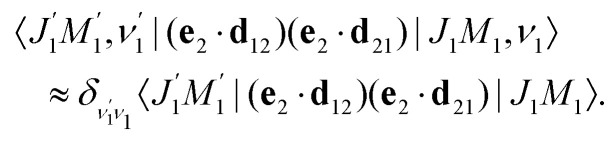
This approximation and the role of vibrations are discussed in greater detail in Section 2.3.3. Eqn (21) and the condition of completeness of the intermediate vibrational states 
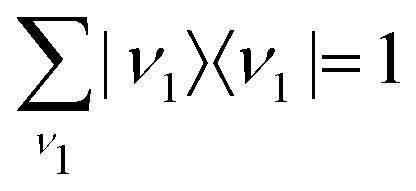
 result in the following expression22
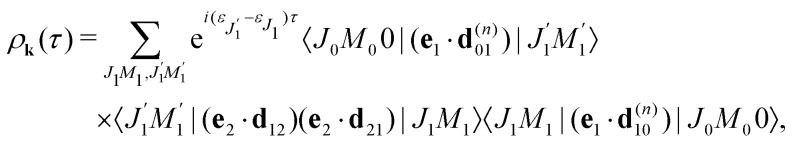
which shows that for short pump and probe X-ray pulses (15) the delay time-dependence is entirely defined by the rotational dynamics in the intermediate state, while the vibrational contribution is entirely cancelled out. The physical background of this important fact is that the rovibrational wave packet has no time to change its shape during the short duration of the pump and probe pulses (see eqn (15), (14), (16) and Section 2.3.3) and it develops only during the delay time between the pulses *τ* ∼ 0.1–1 ps, which is significantly longer than *τ*_d_ ∼ 1 fs < (*ε*_*ν*_1__ + *ε*_*J*_1__)^−1^.

The time-dependence of the probe signal (22) is determined by the spacing between interfering rotational levels 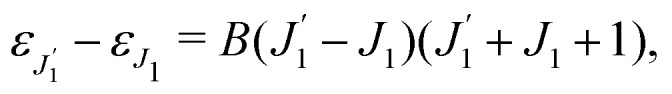 where *B* = 1/2*I* is the rotational constant and *I* is the moment of inertia of the intermediate state.

#### 2.2.2 Thermal population of the ground state rotational levels

We study an ensemble of diatomic molecules in the lowest vibrational level of the ground electronic state. So far, we have neglected the thermal population of rotational states and discussed the XX–PP process starting from the initial rotational state *Y*_*J*_0_*M*_0__(**R̂**). In real system the ground state rotational levels' populations are defined according to the Boltzmann distribution with the Boltzmann constant *k*_B_ and temperature *T*. Accordingly, eqn (22) should be replaced
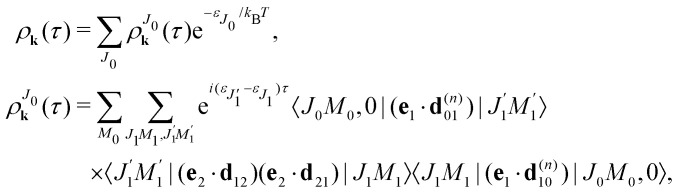
Moreover, according to eqn (18) the probe signal should be integrated over the directions of the photoelectron ejection, which gives us the final expression for the cross section taking into account temperature and photoelectron emission dependence:
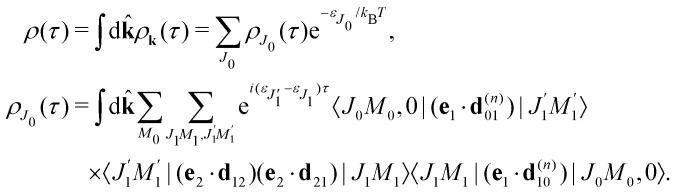


### 2.3 Transition dipole moments and the recoil effect

The classical expression for the recoil angular momentum (6) is not enough to understand the details of the recoil-induced rotation. Here we give the quantum theory of this effect based on the transition dipole moment of photoionization **d**^(*n*)^_10_ which is responsible for the momentum exchange between the molecule and the photoelectron. In order to proceed further we need to compute the dependence of the orientation of the transition dipole moments **d**^(*n*)^_10_ and **d**_21_ on the electron momentum **k** and interatomic radius vector **R**. The transition dipole moment **d**^(*n*)^_10_ of the valence ionization *ψ*_*n*,5σ_ → *ψ*_**k**_ is the product of the atomic prefactor **d**^(*n*)^ and phase factore^∓*iα*_*n*_**k**·**R**^,which describes the recoil effect^10,11,16,19–21^ (see Section I of ESI†). The partial contribution *ψ*_O,5σ_ and *ψ*_C,5σ_ of the 5σ molecular orbital (3) consists of 2s^(*n*)^ and 2p^(*n*)^_σ_ atomic orbitals (see eqn (S10), ESI†). Therefore, **d**^(*n*)^ = *A*_*n*_**k̂** + *B*_*n*_**R̂** + *C*_*n*_(**k̂**·**R̂**)**k̂**, since only 2s^(*n*)^ → *ε*p and 2p^(*n*)^_σ_ → *ε*s, *ε*d ionization channels are allowed. Here and below we use the following notation for the unit vector **k̂** = **k**/*k*. It is worthwhile to note, that the origin of the last two terms in the expression for **d**^(*n*)^ is the 2p^(*n*)^_σ_ ionization channel. Summarizing the above discussion we can write^10,11^ (see Sections I and II of ESI†)

23 **d**^(*n*)^_10_ = **d**^(*n*)^*e*^∓*iα*_*n*_**k**·**R**^, **d**^(*n*)^ = *A*_*n*_**k̂** + *B*_*n*_**R̂** + *C*_*n*_(**k̂**·**R̂**)**k̂**.

Here, one should use e^−*iα*_O_**k**·**R**^ and *e*^*iα*_C_**k**·**R**^ (see Section I of ESI†). The partial transition dipole moment **d**^(*n*)^ is sensitive to the element because the contribution of the atomic orbitals in the 5σ orbital is different for the oxygen and carbon atoms as one can see from the expressions (S17) for *A*_*n*_, *B*_*n*_, and *C*_*n*_ in ESI.†

The dipole moment
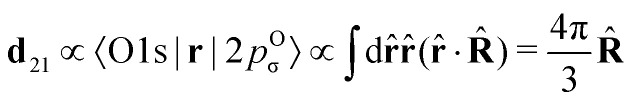
of the probe O1s → 5σ transition is oriented along the unit vector **R̂** = **R**/*R* which is parallel to the molecular axis. The reason for this is that the O1s atomic orbital (AO) is an isotropic function while the 2p^O^_σ_ AO depends on the angle *ϑ* between the radius vector of the electron **r** and **R** as cos *ϑ* ≡ (**r̂**·**R̂**).

To avoid cumbersome expressions, let us consider the case of the 2s^(*n*)^ ionization channel, leading to the following equation for the cross section24
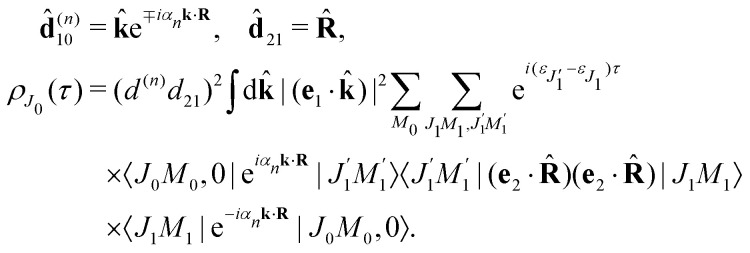
Here *d*^(*n*)^ = |**d**^(*n*)^| and *d*_21_ = |**d**_21_|. The approximation (24) for **d̂**^(*n*)^_10_ is justified for the carbon and oxygen atoms due to small ionization cross section of a 2p electron in comparison with a 2s electron in the region *ε* ≳ 500 eV.^22^ Here and below we use **d**^(*n*)^e^−*iα*_*n*_**k**·**R**^ instead of **d**^(*n*)^e^∓*iα*_*n*_**k**·**R**^. The reason for this is the integration over **k̂** which makes *ρ*_*J*_0__(*τ*) the same for the (−) and (+) signs.

#### 2.3.1 Recoil-induced rotational wave packet

Let us now consider the role of the exponential factor exp(−*iα*_*n*_**k**·**R**) in the expression for the transition dipole moment **d**^(*n*)^_10_(23). This factor comes from the wave function of the photoelectron and it describes the recoil effect.^11,16,19–21^ Indeed, the factor exp(−*iα*_*n*_**k**·**R**) is nothing else than the rotational wave packet created at the instant of the photoionisation due to the recoil effect. This can be seen directly from the well known^23^ Rayleigh expansion of a plane wave25
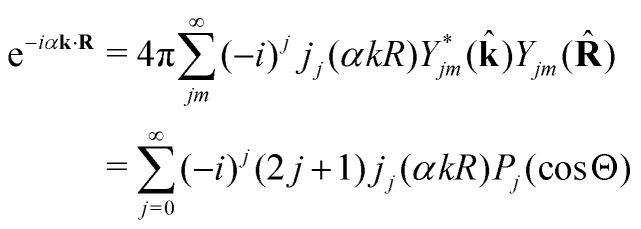
over spherical functions *Y*_*jm*_(**R̂**) which are eigenfunctions of the rotational Hamiltonian *h*^rot^_1_ = *B***J**^2^ of the intermediate state. For the discussion of the wave packet (25) it is more pertinent to examine the distribution *N*_*J*_(*αkR*) over rotational quantum numbers *J*

**Fig. 2:**
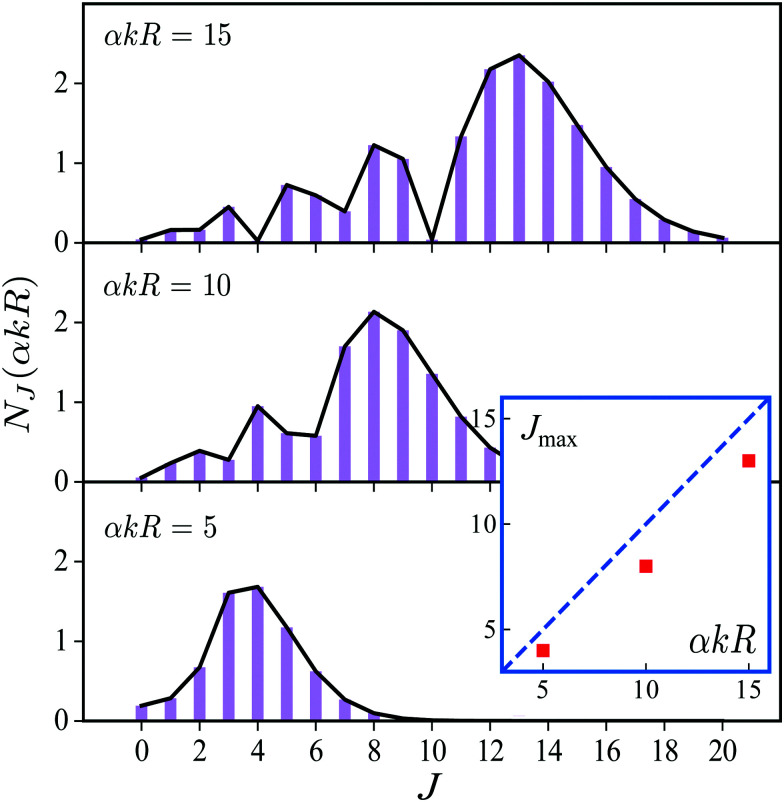
Distribution *N*_*J*_(*αkR*) (26) over *J* in the rotational recoil-induced wave packet (25) for different *αkR*. The red squares in the insert show the dependence of *J*_max_ on the recoil angular momentum *αkR*. Here *J*_max_ is the value of the angular momentum *J* when the distribution *N*_*J*_(*αkR*) takes maximum. The dashed diagonal line in the insert corresponds to the recoil angular momentum *J*_rec_ = *αkR* (6).

26 *N*_*J*_(*αkR*) = (2*J* + 1)|*j*_*J*_(*αkR*)|

shown in Fig. 2. This distribution takes maximum at *J*_max_ ≈ *J*_rec_ = *αkR* (see eqn (6)). That means that the largest contribution to the rotational wave packet (25) at large *αkR* is given by *J*_max_ ≈ *J*_rec_, which shows clearly the physical meaning of the *αkR* term as the recoil angular momentum *J*_rec_(6).

#### 2.3.2 Recoil-induced time structure of the probe signal

We proceed now to obtain an expression for *ρ*_*J*_0__(*τ*) by calculation of the matrix elements in eqn (24). Using eqn (S8) of ESI,† we can write the matrix element as the sum of isotropic and anisotropic contributions
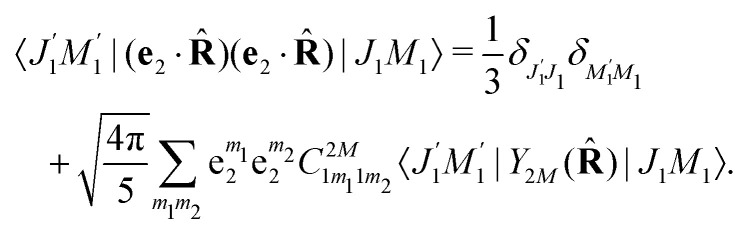
Substitution of this expression in eqn (24) allows us to write the cross section for the probe signal as the sum of isotropic time independent and anisotropic time dependent contributions
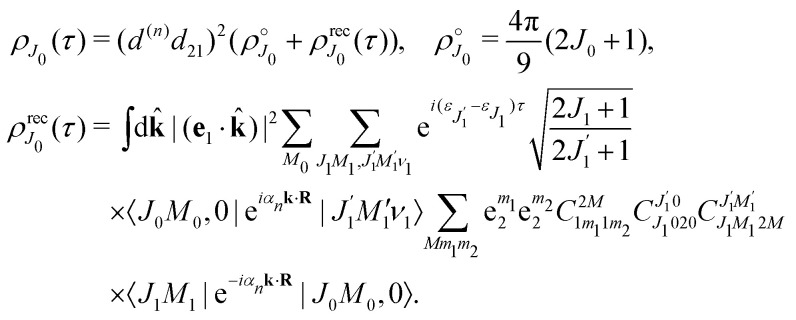
Without losing generality let us orient the polarization vector of the probe field *e*_2_ along the *z*-axis of laboratory frame (*m*_1_ = *m*_2_ = 0). Since *C*_1010_^20^ = 1, we get
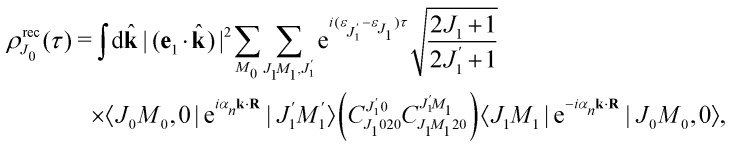
One should notice that the time dependence of 
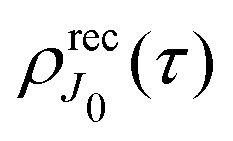
 is solely due to the recoil effect, because 
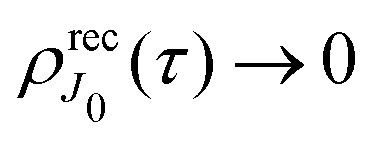
 when *k* → 0:27
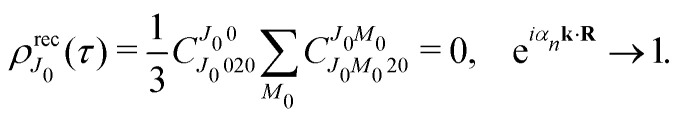
Using the sum rule for the product of three Clebsch–Gordan coefficients^23^ (see eqn (S35) of ESI†), the plane wave expansion (25) and expression (S37) of ESI,† for the matrix element 〈*J*_1_*M*_1_,*ν*_1_|e^−*iα***k**·**R**^|*J*_0_*M*_0_,0〉, we obtain28
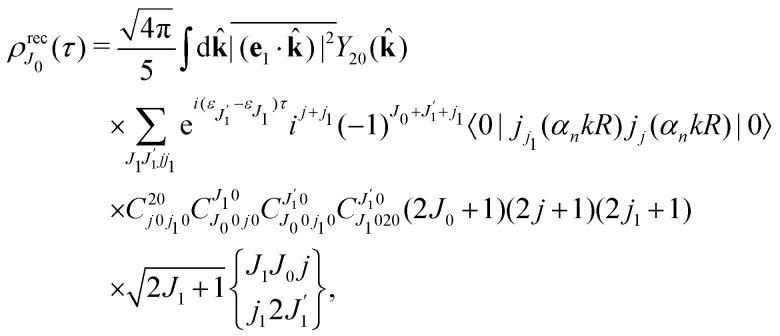
where we use conventional notations for the Clebsch–Gordan coefficients and 6j-symbols, and the overline denotes averaging over the orientation of *e*_1_ around *e*_2_‖*z* axis in laboratory frame.

**Fig. 3:**
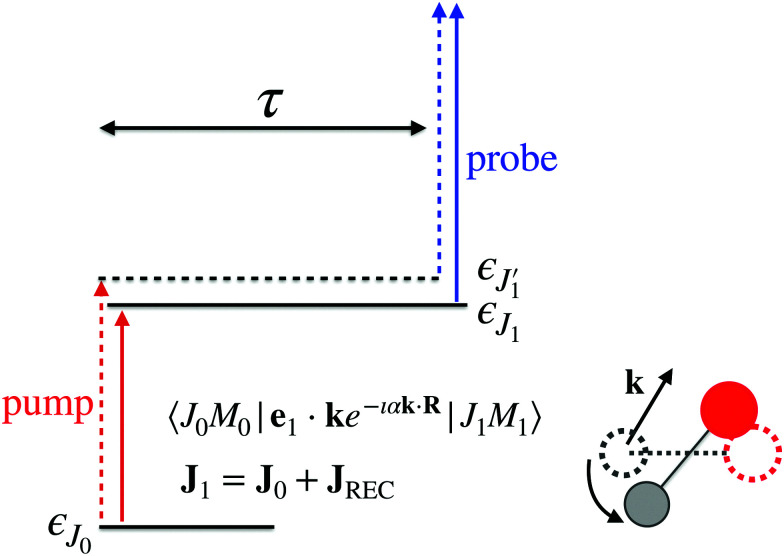
The interference between the rotational levels coherently populated by the pump X-ray pulse results in quantum beats of the time delayed probe X-ray absorption (see eqn (30) and Fig. 4).

Taking into account the small size of the wave function of the lowest vibrational level and performing integration over the directions of the photoelectron ejection
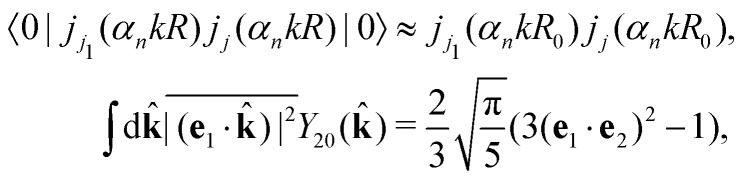
we get finally the following expression for the cross-section29
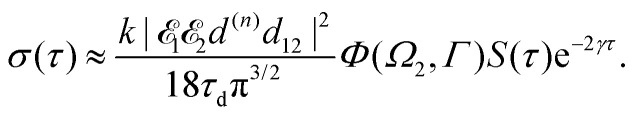
The dependence of the probe signal on the time delay *τ* defined by 
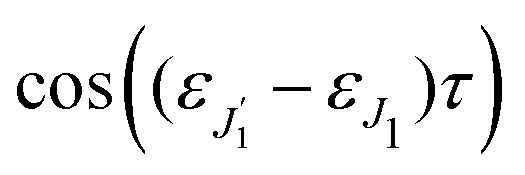
 is given by the function30
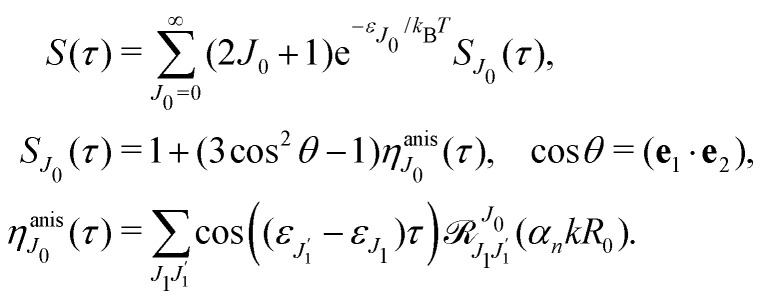
This function depends on the interference between the recoil-induced rotational levels, the thermal population of the ground state rotational levels and the recoil-induced angular momentum. The quantum beats of the anisotropic contribution 
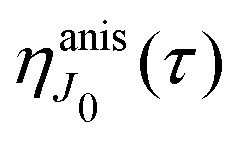
 are caused by the interference between the rotational levels coherently populated by the pump X-ray pulse (Fig. 3).

The recoil-induced coherent excitation of rotational levels is described by the function
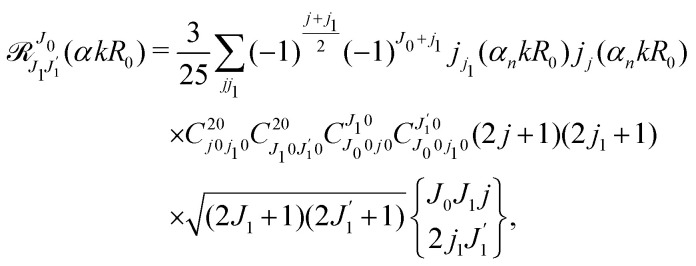
which depends on the angular recoil momentum *J*_rec_ = *αkR*_0_ (6). Here 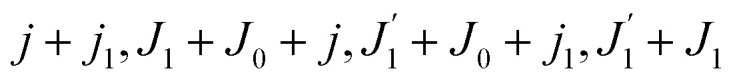 are even. Let us stress, that only the recoil-induced contribution is time-dependent. The recoil-induced contribution is equal to zero when the pump or probe pulse is not polarized 3cos^2^ *θ* − 1 → 0 or *θ* is equal to the magic angle 
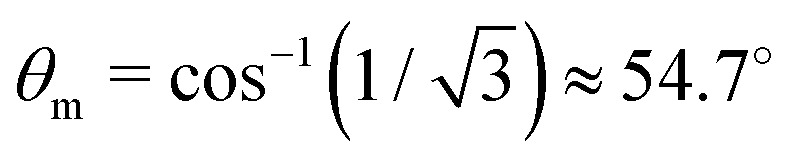
. One should notice that the reason for the polarization dependence of the probe absorption (30) is the structure of the polarization tensor of the two-photon process (see Section IV and eqn (S21) of ESI†).

#### 2.3.3 Role of vibrations

So far, the vibrational dynamics in the intermediated state was neglected starting from eqn (22). One should clarify the limitation of this approximation. The matrix element of the pump transition (see eqn (20))
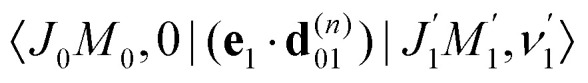
shows that the pump pulse can excite high vibrational states |*ν*_1_〉 with the energies *ε*_*ν*_1__. This may happen due to a large shift between the potential energy surfaces of the ground and ionized states or due to the recoil effect. The larger vibrational energy *ε*_*ν*_1__ can break the short-pulse approximation (eqn (15) and (16)) and, hence, eqn (22). In this case the rovibrational dynamics during the pulse duration can be important and should be taken into account.

However, the rovibrational wave packet can develop also during long delay time between the pulses *τ* ∼ 0.1–1 ps, which is significantly longer than the pulse duration *τ*_d_ ∼ 1 fs. Eqn (22) displays interesting fact – the probe X-ray absorption is not affected by the vibrational dynamics between the pulses. We observe only the rotational dynamics. The formal reason for this is very realistic approximation (21). This approximation can be broken only when the pump pulse excites high vibrational states with a rather large extent of the vibrational wave functions. In this case the R-dependence of the electronic transition dipole moments **d**_12_ can slightly break the discussed approximation. Vibronic coupling can be another reason for violation of the approximation (21).

To conclude, the vibrational dynamics affects the evolution of rotational wave packet in the two cases. First, this happens for long pulses^13,17,18^*τ*_d_*E*_*λ*_*i*__ ≳ 1 when the short-pulse approximation (eqn (15) and (21)) is broken and one should use the strict eqn (14). Second, rotational dynamics could be affected by vibrations also for short pulses (15) if the approximation (16) is not valid.

## 3 Numerical simulations and discussion

Now let us apply the derived eqn (30) to the case-study of the CO molecule with a rotational constant *B* = 1/2*I*, a moment of inertia *I* = *μR*_0_^2^ = 56857.72 a.u. and an equilibrium bond length *R*_0_ = 2.133 a.u. Numerical simulations of eqn (30) are performed using our own FORTRAN code employing equations for the Clebsch–Gordan coefficients and the 6j-symbols.^23^ To ensure the convergence of the results, the upper limit in the sum (30) over the angular momentum *J* is taken to be 50, which is much bigger than the maximal values of the recoil angular momentum *J*^(*n*)^_rec_ (see eqn (6) and Fig. 2).

**Fig. 4:**
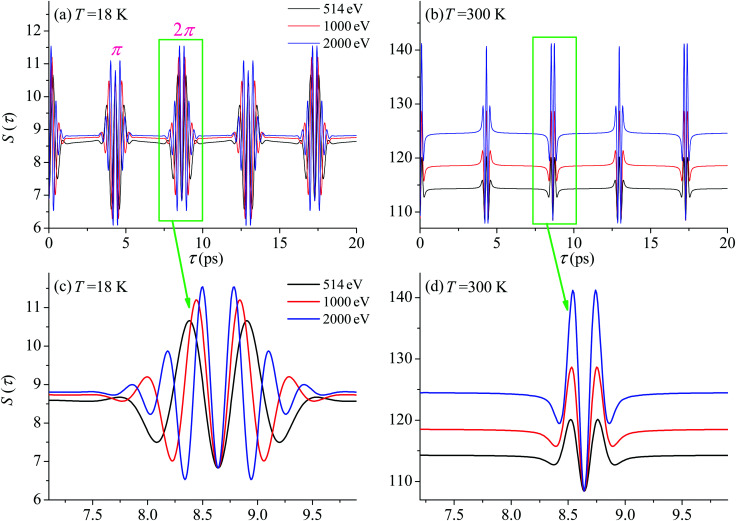
*S*(*τ*) revival structure of the function (30) for the CO molecule computed at the low *T* = 18 K (a and c) and room *T* = 300 K (b and d) temperatures and for different energies of the photoelectron *ε* = 514, 1000 and 2000 eV (see legends); *α*_C_ = 0.571, *θ* = ∠(**e**_1_,**e**_2_) = 0.

First we compute the partial contribution (30) related to the ejection of the valence electron from the carbon atom with *α*_C_ = 0.571. The simulations of the XX–PP process are performed for a low (*T* = 18 K) and room (*T* = 300 K) temperatures and for several frequencies of the pump radiation, corresponding to the following kinetic energies of the photoelectron *ε* = 1, 514, 1000, 2000 eV. The results shown in Fig. 4 indicate that the *S*(*τ*) function for both low and room temperatures has a repeated set of two distinct profiles located at

**Fig. 5:**
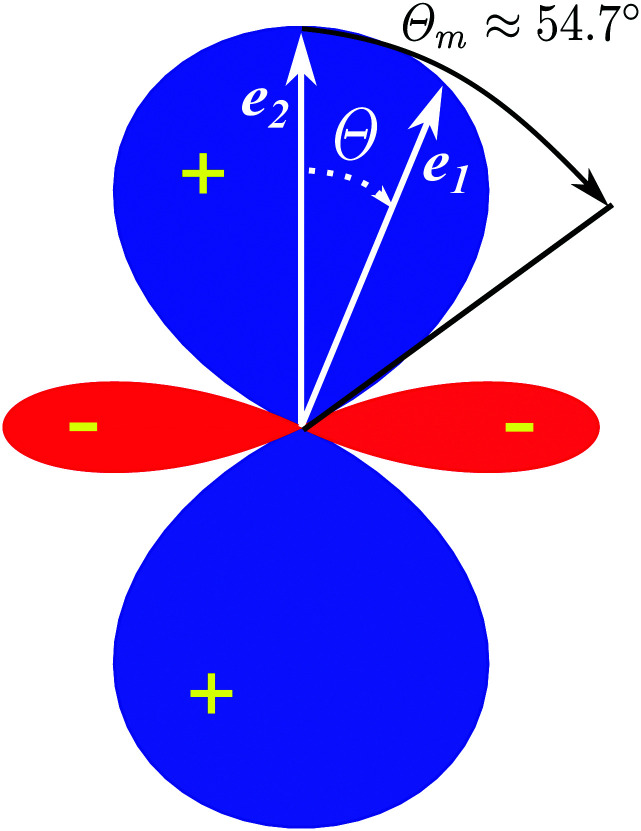
Polar diagram of the polarization function, 3cos^2^ *θ* − 1 (30), *θ* = ∠(**e**_1_,**e**_2_), *θ*_m_ = ∠(**e**_1_,**e**_2_) ≈ 54.7°.

31 *τ* = (4.32 + *n*·8.64) ps, and *τ* = (8.64 + *n*·8.64) ps,

where *n* = 0, 1, 2…. As one can see from Fig. 4 the second rotational recurrence is a copy of the first one, taken with opposite sign. It is not hard to recognize that both of the characteristic times 4.32 ps and 8.64 ps are related to the interference pattern cos((*ε*_1_ − *ε*_0_)*τ*) = cos(*ε*_1_*τ*) = cos(2*Bτ*) of the two lowest rotational levels *J*_0_ = 0 and *J*_0_ = 1 (see eqn (30)). The conditions cos(2*Bτ*) = −1 and cos(2*Bτ*) = +1 give 2*Bτ* = π(1 + 2*n*) and 2*Bτ* = 2π(1 + *n*), respectively. Thus, the evolution of *S*(*τ*) is characterized by two recurrent structures with the same revival time *τ*_rev_
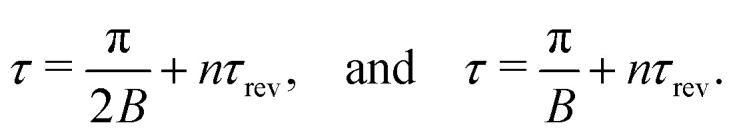
The delay times computed with these equations coincide exactly with the results of the numerical simulations (31). Both profiles revive in time at
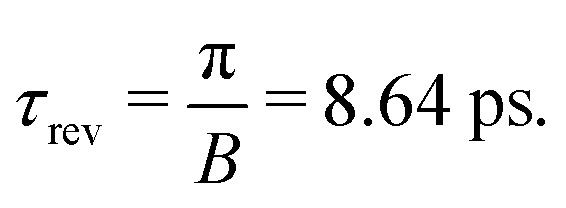
The polarization dependence of the probe signal (30) deserves a special comment. The temporal profile of the revival structures of function *S*(*τ*) get inverted when 3cos^2^ *θ* − 1 < 0 or π − *θ*_m_ ≥ *θ* ≥ *θ*_m_, where *θ*_m_ is the magic angle (see Fig. 5).

**Fig. 6:**
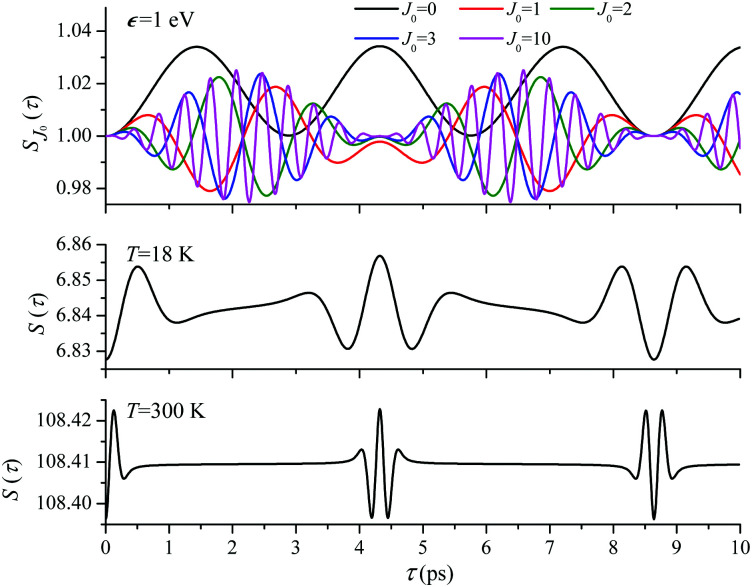
*ε* = 1 eV: *S*(*τ*) function and partial contributions *S*_*J*_0__(*τ*) for T = 18 K and 300 K. See eqn (30). *α*_C_ = 0.571, *θ* = 0. One should notice that in this small energy region the neglected Cohen-Fano interference can be significant (see also Fig. 10).

**Fig. 7:**
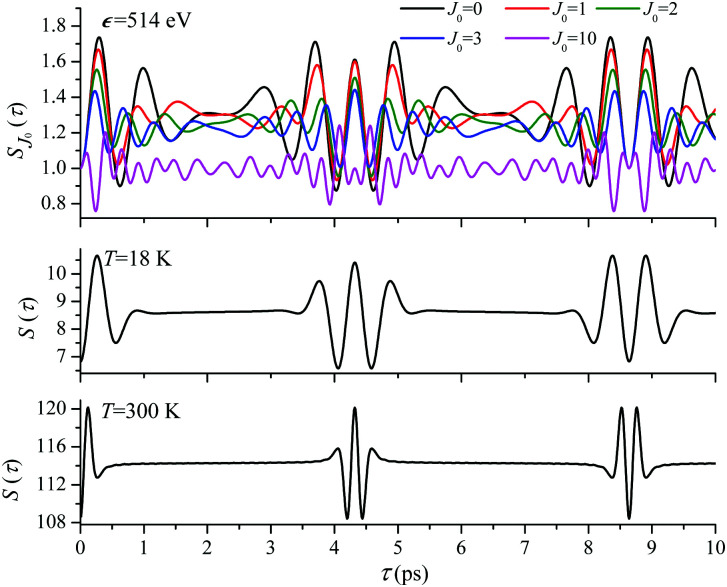
*ε* = 514 eV: *S*(*τ*) function and partial contributions *S*_*J*_0__(*τ*) for *T* = 18 K and 300 K. See eqn (30). *α*_C_ = 0.571, *θ* = 0.

**Fig. 8:**
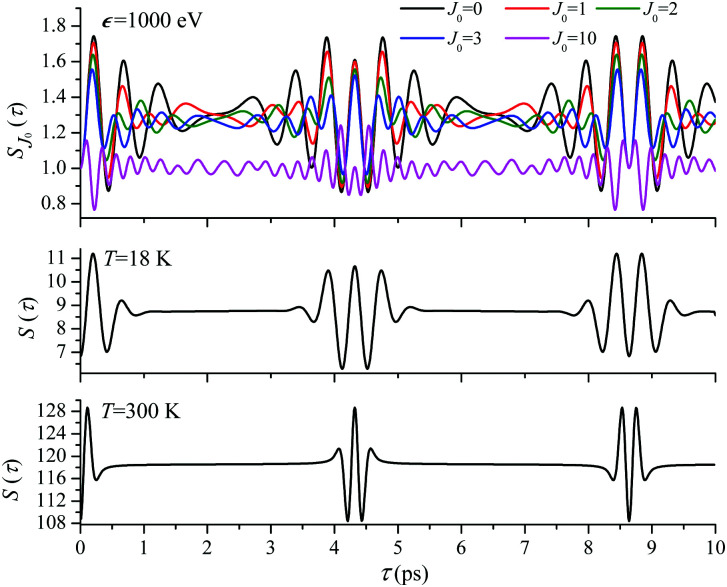
*ε* = 1000 eV: *S*(*τ*) function and partial contributions *S*_*J*_0__(*τ*) for *T* = 18 K and 300 K. See eqn (30). *α*_C_ = 0.571, *θ* = 0.

**Fig. 9:**
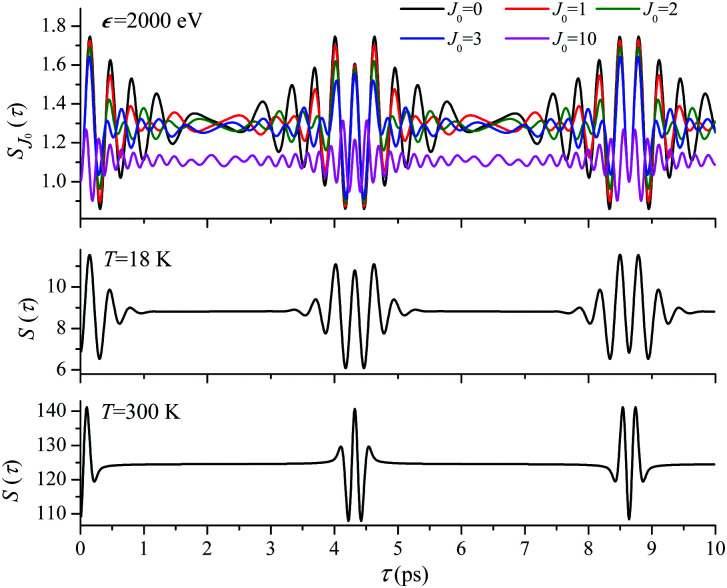
*ε* = 2000 eV: *S*(*τ*) function and partial contributions *S*_*J*_0__(*τ*) for *T* = 18 K and 300 K. See eqn (30). *α*_C_ = 0.571, *θ* = 0.

For the low temperature case the shape of the recoil-induced recurrent structure is very sensitive to the kinetic energy of the photoelectron *ε* (Fig. 4(a) and (c)). The temporal profile gets more peaks with increase of the photoelectron energy (Fig. 4(c)). The shape of the revival structures experiences a narrowing with an increase in temperature (compare Fig. 4(a)–(d)) due to dephasing arising from the contribution of higher initial rotational states *J*_0_, as the number of initial rotational states grows with the temperature as 
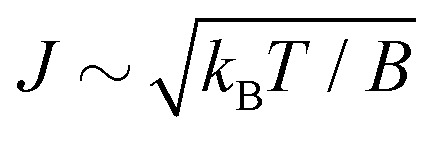
. When the frequency of the pump field is near the ionization threshold the amplitude of the recoil-induced temporal oscillation is small (Fig. 4) due to low value of the photoelectron momentum *k*. As one can see from the upper panel of Fig. 6, when the energy of the photoelectron is small the amplitude of the *S*_*J*_0__(*τ*) modulation (30) is weak and has the same order of magnitude for different *J*_0_. Increasing the photoelectron energy to 514 eV (Fig. 7), 1000 eV (Fig. 8), and 2000 eV (Fig. 9) shows several effects for the recoil-induced recurrent structures. First, the total amplitude of the modulations increases, as compared to the case of *ε* = 1 eV, yet it has a similar value for all high-energy cases (compare Fig. 6–9). Second, the fine profile of the recoil-induced structures becomes more complex with an increase of the photoelectron energy, going from three peaks in the case of *ε* = 514 eV up to seven peaks in the case of *ε* = 2000 eV. Moreover, one can see that the contribution from states with higher angular momenta *J*_0_ becomes stronger, as the photoelectron energy increases (compare upper panels in Fig. 6–9). In the case of high temperature *T* = 300 K (see lower panels in Fig. 6–9) the increase of the photoelectron kinetic energy results only in the increase of the amplitude of the recoil-induced structures, while the fine structure, consisting of three peaks does not change with *ε* variation. The reason for this is a strong dephasing role of thermally populated high initial rotational states (see upper panels of Fig. 6–9).

Until now we focused on analyses of the carbon's contribution to the total cross section *σ*_C_ + *σ*_O_(4). The only difference in the case of the ejection of the valence electron from the oxygen site is the atomic prefactor *d*^(O)^ and the recoil angular momentum *J*^(O)^_rec_ (6) with *α*_O_ = 0.429 which differs from *α*_C_ = 0.571. To shed light on the dependence of the temporal profile on the ionisation site we computed *S*(*τ*) function also for the oxygen ionization channel. The simulations presented in Fig. 11 show very similar temporal profiles for the oxygen and carbon channels. Fig. 11(e) shows that the X-ray absorption probe at 1s core-levels of both the oxygen and carbon atoms of CO only increases the contrast of the total temporal profile (compare panels (c) and (e)). This simulations of panel (e) are done under the assumption an assumption that the partial ionization cross-sections of oxygen and carbon atoms in the CO molecule are the same (|*d*^(O)^|^2^ = |*d*^(C)^|^2^). Thereby we can show that the total signal has a better contrast than the partial contributions even in the case of equal weights of the oxygen and carbon contributions.

**Fig. 10:**
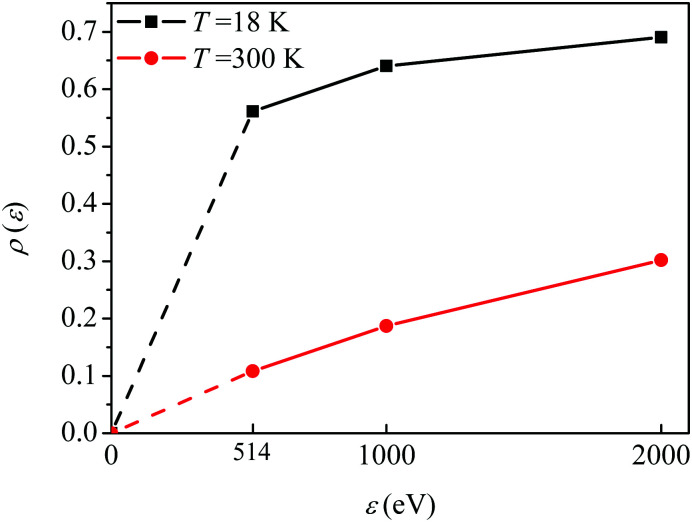
The dependence of the visibility *ρ*(*ε*) (32) of the recoil-induced revivals on the energy of photoelectron *ε*. *α*_C_ = 0.571, *θ* = 0. The reason for *ρ*(0) = 0 is that the recoil-induced population of the rotational levels and, hence, the interference between intermediate rotational levels are absent when the momentum of the photoelectron fulfilled 
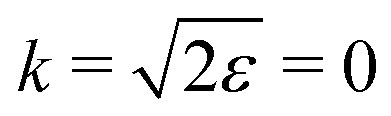
. The dashed lines connecting points *ε* = 514 eV and *ε* = 0 eV indicate that in the region 0 < *ε* ≲ 500 eV the neglected Cohen-Fano interference can be significant.

In order to quantify the visibility of the time-dependence of the recoil-induced structure in the experiment let us compute a relative difference between maximum *S*^max^ and minimum *S*^min^ of *S*(*τ*) function (30), characterising visibility of the effect32
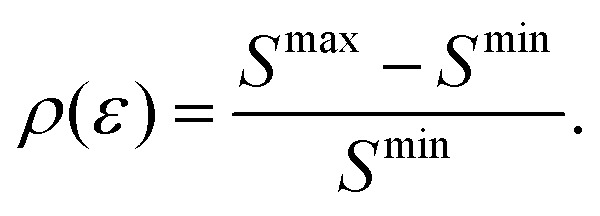
The visibility *ρ*(*ε*) grows monotonously with an increase of the frequency of the pump pulse and hence of the momentum 
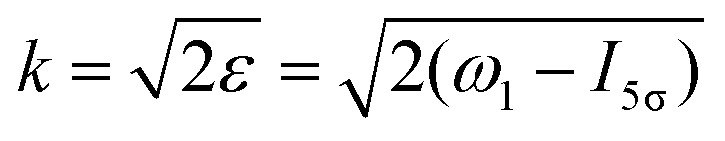
 of photoelectron (see Fig. 10). The visibility is equal to zero (*ρ*(0) = 0) when the photoelectron momentum is zero and thus the recoil is absent (see eqn (27)).

**Fig. 11:**
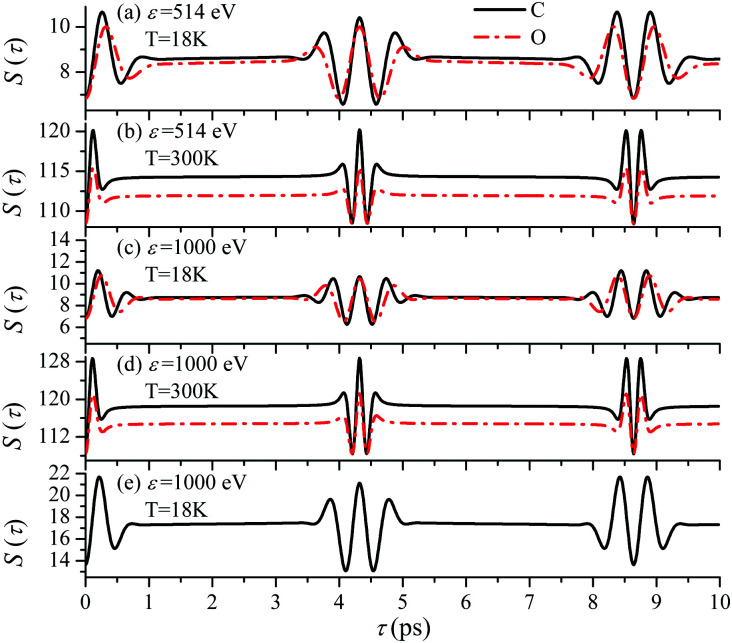
The dependence of the temporal profile *S*(*τ*) (30) on the ionization site n = C, O is defined by the recoil parameter *α*_*n*_*kR*_0_, where *α*_C_ = 0.571, *α*_O_ = 0.429. *θ* = 0. Panel (e) shows the sum *S*(*τ*) = *S*_C_(*τ*) + *S*_O_(*τ*), where *S*_C_(*τ*) and *S*_O_(*τ*) are the *S*(*τ*) functions (30) for carbon and oxygen shown in the panel (c).

The recoil-induced revival structure of the cross section (29) appears without amplitude decay in the case *γ* = 0 when the intermediate |5σ^−1^〉 state is the ground state of the cation CO^+^. However, in general cases the intermediate |5σ^−1^〉 state will be rovibronically excited and broaden due to photoelectron recoil effect, photoexcitation, thermal excitation, *etc.* Therefore, the recurrent structure “is melting” as exp(−*γτ*) (29) due to the finite lifetime of the valence ionized intermediate state.

It is interesting to evaluate also the probe X-ray absorption σ(*τ*) (see eqn (29) and (30)) in the energy domain
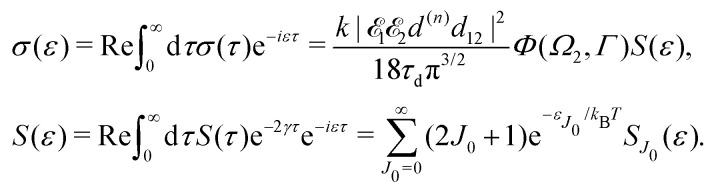
The energy dependence of *σ*(*ε*), defined by the function33
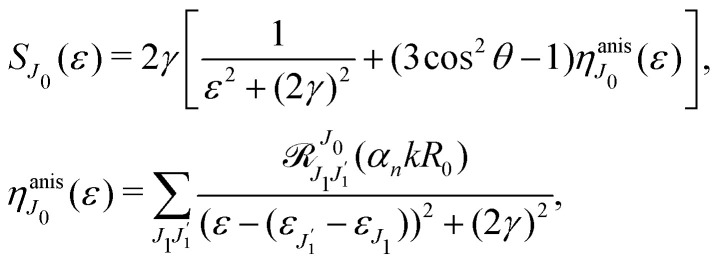
displays the set of narrow resonances with the small lifetime broadening 2*γ* of the intermediate valence ionised state |5σ^−1^〉. One can see that only the anisotropic contribution 
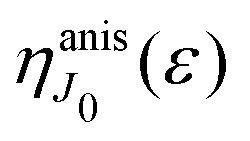
 depends on the rotational structure contrary to the isotropic term which has a maximum at *ε* = 0.

It is worthwhile to note that in the case when the pump and probe pulses are longer or comparable with the vibrational period of the molecule 2π/*ω*_vib_, the nuclear dynamics triggered within the pulse duration will change drastically the profile of the recurrent recoil-induced structures due to the vibrational dynamics and vibrational revivals, which was neglected in the present case of ultrashort X-ray pulses.^24–26^ The period of vibrational revivals *τ*_vib_ = 2π/*ω*_vib_*x*_0_ being inversely proportional to the anharmonicity constant *ω*_vib_*x*_0_ also lies in the ps region.^24–26^ This makes the time structure of rovibrational dynamics for longer pulses more complex and deserving of special investigation. One should notice that the rovibrational dynamics is strongly related to the interference of intermediate rovibrational states.^8,11^

## 4 Summary

We propose a scheme of two-color X-ray pump–probe spectroscopy with short non-overlapping pump and probe pulses. In contrast to stationary X-ray spectroscopy the time-resolved pump–probe X-ray technique gives a unique opportunity to study molecular rotational dynamics, because due to the long rotation period (picosecond range) no high temporal resolution is required. We build up a comprehensive theoretical model describing the change of the temporal absorption profile of the probe pulse due to the recoil-induced rotations triggered by the valence electron photoemission through the interaction with the short pump X-ray pulse. The induced rotational dynamics is seen in the probe signal as two phase shifted recurrent temporal structures. We discuss analytically and study numerically the effect of change of the photoelectron energy and temperature on the fine structure of the recoil-induced X-ray absorption profile. Numerical simulations, based on the case-study of the CO molecule, show narrowing of the temporal profile with an increase of the temperature caused by the thermal dephasing. The recoil-induced temporal profile grows monotonously upon increasing the frequency of the pump X-ray radiation, as corresponding photoelectron energy increase.

We show that the visibility of the effect, computed as the difference between the minimum and maximum of the recoil-induced structure of the probe signal, reaches about 10% for photoelectron kinetic energy 514 eV and increase up to 35% for photoelectron energy 2000 eV. This allows us to conclude on the possibility of a clear experimental observation of the predicted phenomena using the proposed pump–probe scheme, which can be realized at present day X-ray free electron laser facilities, providing pulse durations of less or about 1 fs. The phenomena studied here are not limited to the case of diatomic molecules, but can be also observed in polyatomic molecules. The manifestation of the effect will be modified sufficiently depending on the mass of the system and the molecular orbital emitting the photoelectron.

## Author contributions

F. G. developed the theory and wrote the draft of the article; J. C. L. developed numerical procedures and performed simulations; N. I. performed a part of calculations related to recoil-induced distribution of rotational excitation and prepared a part of figures; V. K. and P. K. discussed the theory, worked with the text and figures; A. F. and M. S. discussed the results of the simulations for experimental observation. All authors participated in the discussion of the results, revision of the manuscript text and figures.

## Conflicts of interest

There are no conflicts to declare.

## Supplementary Material

CP-024-D1CP05000A-s001
