# Supplementary material for: Time-resolved study of recoil-induced rotation by X-ray pump – X-ray probe spectroscopy
Source: Phys Chem Chem Phys. 2022 Feb 24;24(11):6627–38. doi: 10.1039/d1cp05000a (PMC8925299; doi:10.1039/d1cp05000a)
Supplement: CP-024-D1CP05000A-s001 [file CP-024-D1CP05000A-s001.pdf]

Electronic Supplementary Material (ESI) for Physical Chemistry Chemical Physics.
This journal is © the Owner Societies 2022

Supplementary Information for the article "Time-resolved study of recoil-induced rotation by X-ray pump - X-ray probe spectroscopy"

Ji-Cai Liu,[a*] Nina Ignatova,[b] Victor Kimberg,[c,b†] Pavel Krasnov,[b]
Alexander Föhlisch,[d,e] Marc Simon,[f] and Faris Gel'mukhanov[b,c,d]

[a] *Department of Mathematics and Physics, North China Electric Power University, 102206 Beijing, China*
[b] *International Research Center of Spectroscopy and Quantum Chemistry – IRC SQC, Siberian Federal University, 660041 Krasnoyarsk, Russia*
[c] *Department of Theoretical Chemistry and Biology, KTH Royal Institute of Technology, 10691 Stockholm, Sweden*
[d] *Institute for Methods and Instrumentation in Synchrotron Radiation Research FG-ISRR, Helmholtz-Zentrum Berlin für Materialien und Energie Albert-Einstein-Strasse 15, 12489 Berlin, Germany*
[e] *Institut für Physik und Astronomie, Universität Potsdam, Karl-Liebknecht-Strasse 24-25, 14476 Potsdam, Germany*
[f] *Sorbonne Université, CNRS, Laboratoire de Chimie Physique-Matiére et Rayonnement, LCPMR, F-75005, Paris, France*

In Secs. I and II below we compute the transition dipole moments of valence photoionization.

PARTIAL TRANSITION DIPOLE MOMENTS OF PHOTOIONIZATION

According to eq. (5) the HOMO 5σ orbital of CO

$$\psi_{5\sigma}(\mathbf{r}) = \sum_{n=O,C} \psi_{n,5\sigma}(\mathbf{r}-\mathbf{R}_n) \tag{S1}$$

is a coherent superposition of the the wave functions of the oxygen $\psi_{O,5\sigma} \equiv \psi_{O,5\sigma}(\mathbf{r}-\mathbf{R}_O)$ and carbon $\psi_{C,5\sigma} \equiv \psi_{C,5\sigma}(\mathbf{r}-\mathbf{R}_C)$ atoms. Here $\mathbf{r}$ and $\mathbf{R}_n$ are the radius vector of the electron and of the n-th atom. We need to compute the transition dipole of the $5\sigma \to \psi_{\mathbf{k}}$ photoionization

$$\mathbf{d}_{10} = \int d\mathbf{r}\psi^*_{\mathbf{k}}(\mathbf{r})\mathbf{r}\psi_{5\sigma}(\mathbf{r}) = \sum_{n=O,C}\int d\mathbf{r}\ \psi^*_{\mathbf{k}}(\mathbf{r})\mathbf{r}\psi_{n,5\sigma}(\mathbf{r}-\mathbf{R}_n) \approx \sum_{n=O,C}\int d\mathbf{r}\psi^*_{\mathbf{k}}(\mathbf{r})(\mathbf{r}-\mathbf{R}_n)\psi_{n,5\sigma}(\mathbf{r}-\mathbf{R}_n). \tag{S2}$$

Now we are in stage to transform the continuum wave function $\psi_{\mathbf{k}}(\mathbf{r})$ to the same origin as the atomic wave function $\psi_{n,5\sigma}(\mathbf{r}-\mathbf{R}_n)$. First, let us do this using the plane wave approximation

$$\psi_{\mathbf{k}}(\mathbf{r}) \approx \frac{1}{(2\pi)^{3/2}} e^{\imath\mathbf{k}\cdot\mathbf{r}} = \frac{1}{(2\pi)^{3/2}} e^{\imath\mathbf{k}\cdot\mathbf{R}_n} e^{\imath\mathbf{k}\cdot(\mathbf{r}-\mathbf{R}_n)}, \tag{S3}$$

which is quite good approximation for X-ray photoionization of the valence shell. In fact, the plane-wave approximation can be strongly improved [1] by replacing the plane wave $(2\pi)^{-3/2}\exp(\imath\mathbf{k}\cdot(\mathbf{r}-\mathbf{R}_n))$ by solution $\varphi^{(n)}_{\mathbf{k}}(\mathbf{r}-\mathbf{R}_n)$ of the Schrödinger equations in the vicinity of the n-th atom

$$\psi_{\mathbf{k}}(\mathbf{r}) \approx e^{\imath\mathbf{k}\cdot\mathbf{R}_n}\varphi^{(n)}_{\mathbf{k}}(\mathbf{r}-\mathbf{R}_n). \tag{S4}$$

We assumed in eq.(S2) that $\langle\psi_{\mathbf{k}}|\psi_{n,5\sigma}\rangle \approx 0$. This is because $\langle\psi_{\mathbf{k}}|\psi_{n,5\sigma}\rangle \approx e^{-\imath\mathbf{k}\cdot\mathbf{R}_n}\langle\varphi^{(n)}_{\mathbf{k}}(\mathbf{r}-\mathbf{R}_n)|\psi_{n,5\sigma}(\mathbf{r}-\mathbf{R}_n\rangle \approx 0$. Substitution of the wave functions (S1) and (S4) in eq. (S2) results in the following expression for the transition dipole moment of the valence ionization

$$\mathbf{d}_{10} \approx \sum_{n=O,C}\mathbf{d}^{(n)}_{10}, \quad \mathbf{d}^{(n)}_{10} = e^{-\imath\mathbf{k}\cdot\mathbf{R}_n}\mathbf{d}^{(n)}, \tag{S5}$$

* Email: jicailiu@ncepu.edu.cn
† Email: kimberg@kth.se

where

$$\mathbf{d}^{(n)} = \int d\mathbf{r}_n \varphi_{\mathbf{k}}^{(n)*}(\mathbf{r}_n)\mathbf{r}_n\psi_{n,5\sigma}(\mathbf{r}_n), \quad \mathbf{r}_n = \mathbf{r} - \mathbf{R}_n, \tag{S6}$$

is the partial transition dipole moment of the ejection of the photoelectron from the 5σ orbital ($\psi_{n,5\sigma}(\mathbf{r})$) in the vicinity of the n-th atom. Let us proceed further and write $\mathbf{R}_n$ in terms of internuclear radius vector $\mathbf{R} = \mathbf{R}_O - \mathbf{R}_C$ [1]

$$\mathbf{R}_O = \alpha_O\mathbf{R}, \quad \mathbf{R}_C = -\alpha_C\mathbf{R}. \tag{S7}$$

This allows to get

$$\mathbf{d}_{10}^{(O)} = e^{-\imath\alpha_O\mathbf{k}\cdot\mathbf{R}}\mathbf{d}^{(O)}, \quad \mathbf{d}_{10}^{(C)} = e^{\imath\alpha_C\mathbf{k}\cdot\mathbf{R}}\mathbf{d}^{(C)}. \tag{S8}$$

The opposite signs in these exponents are very important for the Cohen-Fano interference because $(\mathbf{d}_{10}^{(O)})^*\mathbf{d}_{10}^{(C)} \propto e^{\imath\mathbf{k}\cdot\mathbf{R}}$ (see Sec. III).

CALCULATION OF $\mathbf{d}^{(n)}$

In this section we show details of derivation of the eq.(23) for $\mathbf{d}^{(n)}$ and clarify the meaning of the coefficients A_n, B_n and C_n. Let us choose the molecular frame with the molecular axis along z-axis

$$\mathbf{R} \parallel z, \quad \theta = \angle(\mathbf{k}, \mathbf{R}). \tag{S9}$$

Using the expansions of the wave functions $\psi_n(\mathbf{r})$ and $\varphi_{\mathbf{k}}^{(n)}(\mathbf{r})$

$$\begin{aligned}\psi_{n,5\sigma}(\mathbf{r}) &= a_{n,5\sigma}R_0^{(n)}(r)Y_{00} + b_{n,5\sigma}R_1^{(n)}(r)Y_{1z}(\hat{\mathbf{r}}),\\ \varphi_{\mathbf{k}}^{(n)}(\mathbf{r}) &= \sum_{lm}\chi_{k,l}^{(n)}(r)Y_{lm}^*(\hat{\mathbf{k}})Y_{lm}(\hat{\mathbf{r}}) = \sum_{lm}\chi_{k,l}^{(n)}(r)Y_{lm}(\hat{\mathbf{k}})Y_{lm}^*(\hat{\mathbf{r}})\end{aligned} \tag{S10}$$

over spherical harmonics $Y_{lm}(\hat{\mathbf{r}})$ we write $\mathbf{d}^{(n)}$ (S6)

$$\begin{aligned}\mathbf{d}^{(n)} &= a_{n,5\sigma}\sum_{lm}Y_{lm}^*(\hat{\mathbf{k}})P_{0l}^{(n)}\langle Y_{00}|\hat{\mathbf{r}}|Y_{lm}\rangle + b_{n,5\sigma}\sum_{lm}Y_{lm}^*(\hat{\mathbf{k}})P_{1l}^{(n)}\langle Y_{1z}|\hat{\mathbf{r}}|Y_{lm}\rangle,\\ P_{lL}^{(n)} &= \int_0^\infty drr^3\chi_{k,l}^{(n)*}(r)R_L^{(n)}(r), \quad L = 0, 1.\end{aligned} \tag{S11}$$

in terms of the radial integrals $P_{Ll}^{(n)}$ and the spherical functions $Y_{lm}(\hat{\mathbf{k}})$. It is convenient to use the expansion of $\hat{\mathbf{r}}$ over the real spherical functions and the relationship between real ($Y_{1\mu}(\hat{\mathbf{r}})$, $\mu = x, y, z$) and complex ($Y_{1m}(\hat{\mathbf{r}})$, $m = 0, \pm 1$) spherical function [2]

$$\begin{aligned}\hat{\mathbf{r}} &= \sum_{\mu=x,y,z}\hat{\mathbf{r}}_\mu\mathbf{e}_\mu = \sqrt{\frac{4\pi}{3}}\sum_{\mu=x,y,z}Y_{1\mu}(\hat{\mathbf{r}})\mathbf{e}_\mu,\\ Y_{1x} &= \frac{Y_{1-1} - Y_{11}}{\sqrt{2}}, \quad Y_{1y} = \imath\frac{Y_{11} + Y_{1-1}}{\sqrt{2}}, \quad Y_{1z} = Y_{10}.\end{aligned} \tag{S12}$$

Here $\mathbf{e}_\mu$ is the unit vector along the μ-th axis. Putting together this, the expression for the matrix element [2]

$$\langle Y_{1z}|Y_{1m'}|Y_{lm}\rangle = \delta_{m',-m}(-1)^m\frac{1}{\sqrt{4\pi}}\left[\delta_{l,0} + \sqrt{\frac{4-m^2}{5}}\delta_{l,2}\right] \tag{S13}$$

and

$$\hat{\mathbf{k}}_x = \sin\theta\cos\varphi, \quad \hat{\mathbf{k}}_y = \sin\theta\sin\varphi, \quad \hat{\mathbf{k}}_z = \cos\theta, \quad \hat{\mathbf{R}} = \mathbf{e}_z \tag{S14}$$

we obtain the following expression for the transition dipole moment of our interest

$$\mathbf{d}_x^{(n)} = \frac{1}{\sqrt{4\pi}} a_{n,5\sigma} P_{10}^{(n)} \hat{\mathbf{k}}_x + b_{n,5\sigma} P_{21}^{(n)} \frac{1}{2}\sqrt{\frac{3}{\pi}} (\hat{\mathbf{k}}\cdot\hat{\mathbf{R}})\hat{\mathbf{k}}_x$$

$$\mathbf{d}_y^{(n)} = \frac{1}{\sqrt{4\pi}} a_{n,5\sigma} P_{10}^{(n)} \hat{\mathbf{k}}_y + b_{n,5\sigma} P_{21}^{(n)} \frac{1}{2}\sqrt{\frac{3}{\pi}} (\hat{\mathbf{k}}\cdot\hat{\mathbf{R}})\hat{\mathbf{k}}_y, \tag{S15}$$

$$\mathbf{d}_z^{(n)} = \frac{1}{\sqrt{4\pi}} a_{n,5\sigma} P_{10}^{(n)} \hat{\mathbf{k}}_z + b_{n,5\sigma} P_{01}^{(n)} \frac{1}{2\sqrt{3\pi}} + b_{n,5\sigma} P_{21}^{(n)} \frac{1}{2\sqrt{3\pi}} (3(\hat{\mathbf{k}}\cdot\hat{\mathbf{R}})^2 - 1),$$

These components of $\mathbf{d}^{(n)}$ and eq.(S14) allows to write the transition dipole moment $\mathbf{d}^{(n)} = \sum_{\mu=x,y,z} \mathbf{d}_\mu^{(n)} \mathbf{e}_\mu$ in the invariant form valid in any frame

$$\mathbf{d}^{(n)} = \frac{a_{n,5\sigma}}{\sqrt{4\pi}} P_{10}^{(n)} \hat{\mathbf{k}} + \frac{b_{n,5\sigma}}{2\sqrt{3\pi}} (P_{01}^{(n)} - P_{21}^{(n)}) \hat{\mathbf{R}} + \frac{b_{n,5\sigma}}{2}\sqrt{\frac{3}{\pi}} P_{21}^{(n)} (\hat{\mathbf{k}}\cdot\hat{\mathbf{R}})\hat{\mathbf{k}}. \tag{S16}$$

The obtained equation explains the expression (23) for $\mathbf{d}^{(n)}$ and clarifies the meaning of the coefficients A_n, B_n and C_n in eq.(23) of the main text

$$A_n = \frac{a_{n,5\sigma}}{\sqrt{4\pi}} P_{10}^{(n)}, \quad B_n = \frac{b_{n,5\sigma}}{2\sqrt{3\pi}} (P_{01}^{(n)} - P_{21}^{(n)}), \quad C_n = b_{n,5\sigma}\sqrt{\frac{3}{4\pi}} P_{21}^{(n)}. \tag{S17}$$

COHEN-FANO INTERFERENCE

In the present section we explain the partition of the ionization cross-section (4) in three contributions and explain why the interference term σ_{int} (5) is negligibly small in X-ray ionization of valence electrons. Due to the coherence of the oxygen and carbon contributions in the 5σ molecular orbital, one can expect two-center interference of the $\psi_O \to \psi_{\mathbf{k}}$ and $\psi_C \to \psi_{\mathbf{k}}$ ionization channels. Let as compute the 5σ ionization cross section using eq. (S5)

$$\begin{aligned}\sigma_{5\sigma} &\propto \frac{1}{4\pi}\int d\hat{\mathbf{R}} |\mathbf{d}_{10}|^2 = \frac{1}{4\pi}\int d\hat{\mathbf{R}} \left| \sum_{n=O,C} e^{-\imath \mathbf{k}\cdot\mathbf{R}_n} \mathbf{d}^{(n)} \right|^2 \\ &= \sum_{n=O,C} \left|\mathbf{d}^{(n)}\right|^2 + 2\mathrm{Re}\left(\mathbf{d}^{(O)*}\mathbf{d}^{(C)} \frac{1}{4\pi}\int d\hat{\mathbf{R}} e^{\imath \mathbf{k}\cdot\mathbf{R}}\right) \\ &= \sum_{n=O,C} \left|\mathbf{d}^{(n)}\right|^2 + 2\mathrm{Re}\left(\mathbf{d}^{(O)*}\mathbf{d}^{(C)}\right) \frac{\sin kR}{kR}.\end{aligned} \tag{S18}$$

Here (but not in the main text) we neglected rather weak dependence on $\hat{\mathbf{R}}$ of the direct terms $\left|\mathbf{d}^{(n)}\right|^2$ in comparison with the strong $\hat{\mathbf{R}}$ dependence of the interference factor $\exp(\imath \mathbf{k}\cdot\mathbf{R})$. Thus we get eqs. (4) and (5), where

$$\sigma_{5\sigma} = \sigma_O + \sigma_C + \sigma_{int}, \quad \sigma_n \propto \left|\mathbf{d}^{(n)}\right|^2, \quad \sigma_{int} \propto 2\mathrm{Re}\left(\mathbf{d}^{(O)*}\mathbf{d}^{(C)}\right) \frac{\sin kR}{kR}. \tag{S19}$$

One can see that the Cohen-Fano (CF) interference term σ_{int} [3] is comparable with the direct terms σ_n when the photon frequency is close to the ionization threshold, where $\sin(kR)/kR \approx 1$ because here the momentum k is small. However, $\sigma_{int} \propto (kR)^{-1}$ is strongly suppressed in the valence X-ray ionization due to the large momentum of the photoelectron and because of random orientation of free molecules.

POLARIZATION TENSOR

To give more insight in the polarization dependence of the probe X-ray absorption (see eqs. (29) and (30) of the main text) here we provide in-deep physical analysis, starting from eq.(24) of the main text:

$$\begin{aligned}\varrho_{J_0}(\tau) &= (d^{(n)} d_{21})^2 \int d\hat{\mathbf{k}} \sum_{M_0} \sum_{J_1 M_1, J_1' M_1'} e^{\imath(\epsilon_{J_1'} - \epsilon_{J_1})\tau} \\ &\times \langle J_0 M_0, 0| e^{\imath \alpha_n \mathbf{k}\cdot\mathbf{R}} | J_1' M_1'\rangle \langle J_1' M_1'| (\mathbf{e}_1\cdot\hat{\mathbf{k}})^2 (\mathbf{e}_2\cdot\hat{\mathbf{R}})^2 | J_1 M_1\rangle \langle J_1 M_1 | e^{-\imath \alpha_n \mathbf{k}\cdot\mathbf{R}} | J_0 M_0, 0\rangle.\end{aligned} \tag{S20}$$

Apparently, $\varrho_{J_0}(\tau)$ depends only on the angle θ between the polarization vectors $\mathbf{e}_1$ and $\mathbf{e}_2$. The reason for this is the random molecular orientation in rotational states J_0 (the formal reason for this is integration over $\hat{\mathbf{R}}$ in matrix elements and summations over all projections of angular momentum M) and integration over all directions of ejection of the photoelectron. This means that $\varrho_{J_0}(\tau)$ exactly coincides with $\overline{\varrho_{J_0}(\tau)}$ averaged over all orientations of the pair ($\mathbf{e}_1$, $\mathbf{e}_2$) with fixed angle θ between them. Following Ref. [4] let us perform this orientational averaging of the product of the cartesian coordinates of the polarization vectors $\mathbf{e}_1$ and $\mathbf{e}_2$ (see Sec. IV A)

$$\overline{e_{1i}e_{1j}e_{2k}e_{2l}} = \frac{1}{9}\left[\delta_{ij}\delta_{kl} + \frac{(3\cos^2\theta - 1)}{5}\left(\frac{3(\delta_{ik}\delta_{jl} + \delta_{il}\delta_{jk})}{2} - \delta_{ij}\delta_{kl}\right)\right]. \tag{S21}$$

Here overline denotes the averaging over orientations of the pair ($\mathbf{e}_1$, $\mathbf{e}_2$) with fixed angle $\theta = \angle(\mathbf{e}_1, \mathbf{e}_2)$. As we will see below, the anisotropic term ($\propto (3\cos^2\theta - 1)$ in the polarization tensor (S21) is the reason for the polarization dependence of the probe X-ray absorption (see eqs. (29) and (30) of the main text). Eq. (S21) results in the following expression [4, 5]

$$\overline{(\mathbf{e}_1\cdot\hat{\mathbf{k}})^2(\mathbf{e}_2\cdot\hat{\mathbf{R}})^2} = \frac{1}{9}\left[1 + \frac{1}{5}(3\cos^2\theta - 1)(3(\hat{\mathbf{k}}\cdot\hat{\mathbf{R}})^2 - 1)\right]. \tag{S22}$$

The replacement $(\mathbf{e}_1\cdot\hat{\mathbf{k}})^2(\mathbf{e}_2\cdot\hat{\mathbf{R}})^2$ in eq. (S20) by $\overline{(\mathbf{e}_1\cdot\hat{\mathbf{k}})^2(\mathbf{e}_2\cdot\hat{\mathbf{R}})^2}$ gives the following expression for

$$\begin{aligned}\varrho_{J_0}(\tau) = \overline{\varrho_{J_0}(\tau)} = \frac{4\pi}{9}(d^{(n)}d_{21})^2(2J_0+1)\Big[1 + \frac{(3\cos^2\theta - 1)}{10\pi(2J_0+1))}\int d\hat{\mathbf{k}}\sum_{M_0}\sum_{J_1M_1,J_1'M_1'} e^{\imath(\epsilon_{J_1'}-\epsilon_{J_1})\tau} \\ \times\langle J_0M_0,0|e^{\imath\alpha_n\mathbf{k}\cdot\mathbf{R}}|J_1'M_1'\rangle\langle J_1'M_1'|P_2(\hat{\mathbf{k}}\cdot\hat{\mathbf{R}})|J_1M_1\rangle\langle J_1M_1|e^{-\imath\alpha_n\mathbf{k}\cdot\mathbf{R}}|J_0M_0,0\rangle\Big]\end{aligned} \tag{S23}$$

which is the sum of the isotropic time-independent and anisotropic time-dependent $\propto (3\cos^2\theta - 1)$ contributions. Here $P_2(x) = (3x^2 - 1)/2$ is the Legendre polynomial of order 2. This expression explains the structure of the final eq. (30) of the article. One can show that this equation finally results in eqs.(29) and (30) of the main text. We would like to point out that the polarization dependence of the probe absorption (eqs.(29) and (30) of the main text) is nothing special. The same polarization tensor (eqs. (S22) and (S21)) describes the polarization properties of other resonant two-photon processes, for example resonant inelastic X-ray scattering by free molecules [4, 5].

Derivation of eq.(S21)

In general case, the polarization tensor $\overline{e_{1i}e_{1j}e_{2k}e_{2l}}$ of rang 4 can be constructed as linear combination of three possible combinations of the products of two Kronecker deltas $\delta_{ij}\delta_{kl}$

$$\overline{e_{1i}e_{1j}e_{2k}e_{2l}} = A\delta_{ij}\delta_{kl} + B\delta_{ik}\delta_{jl} + C\delta_{il}\delta_{jk}. \tag{S24}$$

To find the unkown coefficients A, B, and C we should use the following special sums

$$\begin{aligned} i=j,\ k=l: &\quad \sum_{ik}\overline{e_{1i}e_{1i}e_{2k}e_{2k}} \equiv 1 = 9A + 3B + 3C, \\ i=k,\ j=l &\quad \sum_{ij}\overline{e_{1i}e_{2i}e_{1j}e_{2j}} \equiv (\mathbf{e}_1\cdot\mathbf{e}_2)^2 = 3A + 9B + 3C, \\ i=l,\ j=k: &\quad \sum_{ik}\overline{e_{1i}e_{2i}e_{1k}e_{2k}} \equiv (\mathbf{e}_1\cdot\mathbf{e}_2)^2 = 3A + 3B + 9C. \end{aligned} \tag{S25}$$

Solution of these equations

$$A = \frac{2 - \cos^2\theta}{15}, \quad B = C = \frac{3\cos^2\theta - 1}{30} \tag{S26}$$

results in eq.(S21). It is interesting to apply obtained result to the special case of the same polarization vectors $\mathbf{e}_1 = \mathbf{e}_2 = \mathbf{e}$. Since now $\cos\theta = 1$ and $A = B = C = 1/15$ we get well known result [6]

$$\overline{e_ie_je_ke_l} = \frac{1}{15}(\delta_{ij}\delta_{kl} + \delta_{ik}\delta_{jl} + \delta_{il}\delta_{jk}). \tag{S27}$$

DETAILS OF THE PROBE SIGNAL CALCULATIONS

In this section we derive eqs. (10) and (18) of the main text. Substitution of the solution (9) in expression (8) for the probability of absorption of the second pulse (see main text) results in the following expression

$$\sigma_{\mathbf{k}}(\tau,t) = 2\sum_{\lambda_1,\lambda_2,\lambda_1'} \mathrm{Re}\Big[\int_{-\infty}^{t} dt_3 e^{\gamma t_3}\langle\lambda_0|G_{01}(t_3)|\lambda_1\rangle e^{-\imath(\omega_{10}+E_{\lambda_1}-\omega_1)t_3}\langle\lambda_1|G_{12}(t-\tau)|\lambda_2\rangle e^{-(\Gamma+\gamma)t}e^{\imath(\omega_2-\omega_{21}-E_{\lambda_2}+E_{\lambda_1})t}$$
$$\times\int_{-\infty}^{t} dt_1\Big\{\langle\lambda_2|G_{21}(t_1-\tau)|\lambda_1'\rangle e^{\imath(\omega_{21}+E_{\lambda_2}-E_{\lambda_1'}-\omega_2)t_1}e^{\Gamma t_1}e^{-\gamma t_1}\int_{-\infty}^{t_1} dt_2 e^{\gamma t_2}\langle\lambda_1'|G_{10}(t_2)|\lambda_0\rangle e^{\imath(\omega_{10}+E_{\lambda_1'}-\omega_1)t_2}\Big\}\Big]. \quad \text{(S28)}$$

This equation is too cumbersome. We wish to rewrite it in terms of the evolution operators $e^{-\imath H_1\tau}$ and $e^{-\imath H_2\tau}$ which makes expression for $\sigma_{\mathbf{k}}(\tau,t)$ not only significantly simpler but also puts forward the dynamics of the nuclear wave packet between the pulses. Using condition of completeness

$$\sum_{\lambda_m}|\lambda_m\rangle\langle\lambda_m| = 1, \quad \sum_{\lambda_m}|\lambda_m\rangle\langle\lambda_m|e^{\imath E_{\lambda_m}t} = e^{\imath H_m t}, \quad \text{(S29)}$$

we eliminate the sum over the quantum states $\lambda_1, \lambda_2, \lambda_1'$ in eq. (S28) and obtain the expression for $\sigma_{\mathbf{k}}(\tau,t)$ in compact operator form (see eq. (10) in the main text).

Let us now perform integration over time for the short non-overlapping pump and probe pulses using eqs. (12) and (16) of the main text. Taking into account that $G_{10}(t) = \mathcal{E}_1(t)(\mathbf{e}_1\cdot\mathbf{d}_{10}^{(n)})/2$ and $G_{21}(t) = \mathcal{E}_2(t)(\mathbf{e}_2\cdot\mathbf{d}_{21})/2$, one can rewrite eq. (16) as

$$\sigma_{\mathbf{k}}(\tau) = \frac{1}{8}e^{-2\gamma\tau}\mathrm{Re}\Big\{\langle\lambda_0|(\mathbf{e}_1\cdot\mathbf{d}_{01}^{(n)})e^{\imath H_1\tau}(\mathbf{e}_2\cdot\mathbf{d}_{12})e^{-\imath H_2\tau}e^{\imath H_2\tau}(\mathbf{e}_2\cdot\mathbf{d}_{21})e^{-\imath H_1\tau}(\mathbf{e}_1\cdot\mathbf{d}_{10}^{(n)})|\lambda_0\rangle$$
$$\times\Big|\int_{-\infty}^{\infty} dt\mathcal{E}_1(t)e^{-\imath\Omega_1 t}\Big|^2\int_{-\infty}^{\infty} dt\int_{-\infty}^{t} dt_1\mathcal{E}_2^*(t)\mathcal{E}_2(t_1)e^{\imath(\Omega_2+\imath\Gamma)(t-t_1)}\Big\}. \quad \text{(S30)}$$

We now turn to the calculation of the integrals in this equation. Assuming the Gaussian temporal envelops (17) for the pump and probe pulses the integrals in eq. (S30) can be easily computed

$$\Big|\int_{-\infty}^{\infty} dt\mathcal{E}_1(t)e^{-\imath\Omega_1 t}\Big|^2 = 2|\mathcal{E}_1|^2e^{-(\Omega_1\tau_d)^2}, \quad \text{(S31)}$$
$$\int_{-\infty}^{\infty} dt\int_{-\infty}^{t} dt_1\mathcal{E}_2^*(t)\mathcal{E}_2(t_1)e^{\imath(\Omega_2+\imath\Gamma)(t-t_1)} = \frac{|\mathcal{E}_2|^2}{\pi\tau_d^2}\int_{-\infty}^{\infty} dt\int_{-\infty}^{t} dt_1 e^{-(t^2+t_1^2)/2\tau_d^2}e^{\imath(\Omega_2+\imath\Gamma)(t-t_1)}$$
$$= \frac{|\mathcal{E}_2|^2}{\pi\tau_d^2}\int_{-\infty}^{\infty} dt_+e^{-t_+^2/2\tau_d^2}\int_{0}^{\infty} dt_-e^{-t_-^2/2\tau_d^2}e^{\imath\sqrt{2}(\Omega_2+\imath\Gamma)t_-} = 2|\mathcal{E}_2|^2\Psi(\Omega_2,\Gamma),$$

where

$$\Psi(\Omega_2,\Gamma) = \frac{1}{\tau_d\sqrt{2\pi}}\int_{0}^{\infty} dte^{-t^2/2\tau_d^2}e^{\imath\sqrt{2}(\Omega_2+\imath\Gamma)t}, \quad \text{(S32)}$$

is the complex Voigt function and $t_\pm = (t\pm t_1)/\sqrt{2}$. Substitution of the eqs. (S31) in eq. (S30) results in the expression

$$\sigma_{\mathbf{k}}(\tau) = \frac{|\mathcal{E}_1\mathcal{E}_2|^2}{2}e^{-2\gamma\tau}e^{-(\Omega_1\tau_d)^2}\Phi(\Omega_2,\Gamma) \quad \text{(S33)}$$
$$\times\langle J_0M_0,0|(\mathbf{e}_1\cdot\mathbf{d}_{01}^{(n)})e^{\imath H_1\tau}(\mathbf{e}_2\cdot\mathbf{d}_{12})(\mathbf{e}_2\cdot\mathbf{d}_{21})e^{-\imath H_1\tau}(\mathbf{e}_1\cdot\mathbf{d}_{10}^{(n)})|J_0M_0,0\rangle.$$

Here $\Phi(\Omega_2,\Gamma)=\text{Re}\ \Psi(\Omega_2,\Gamma)$ (see eq. (19) of the main text). By integrating $\sigma_{\mathbf{k}}(\tau)$ over the photoelectron momentum $\mathbf{k}$

$$\sigma(\tau)=2\int\frac{d\mathbf{k}}{(2\pi)^3}\sigma_{\mathbf{k}}(\tau), \tag{S34}$$

one obtains the absorption cross section of the probe X-ray pulse (eq. (18) of the main text). In agreement with the intuition the dynamics of the nuclear wave packet is fully defined by the evolution $e^{-\imath H_1\tau}$ in the pumped state in the case of short probe pulse. The formal reason for this is that in eq. (S30) $e^{-\imath H_2\tau}e^{\imath H_2\tau}=1$, while the physical explanation is that the evolution in the final state does not affect the studied process due to short X-ray pulses (see eq. (15) and related discussion in the main text).

SPHERICAL FUNCTIONS AND CLEBSCH-GORDAN COEFFICIENTS

Here we collect some important equations of the quantum theory of angular momentum [2] used in the main text. To get the final expression (28) for $\varrho^{\text{rec}}_{J_0}(\tau)$ we use the sum rule for the product of three Clebsch-Gordan coefficients [2]

$$\sum_{M_0M_1}C^{J_1M_1}_{J_0M_0jm}C^{J_1'M_1}_{J_0M_0j_1m}C^{J_1'M_1}_{J_1M_120}=(-1)^{J_0+j+J_1'}\frac{(2J_1'+1)\sqrt{2J_1+1}}{\sqrt{2j_1+1}}C^{j_1m}_{jm20}\left\{\begin{array}{c}J_1J_0j\\ j_12J_1'\end{array}\right\}. \tag{S35}$$

Here we use the conventional notations for Clebsch-Gordan coefficients and 6j-symbols [2]. Let us write down few equations [2] which are needed for the derivation performed in Sec. 2.3.2 of the main text:

$$\begin{aligned}&(\mathbf{e}_2\cdot\hat{\mathbf{R}})(\mathbf{e}_2\cdot\hat{\mathbf{R}})=\frac{1}{3}+\sqrt{\frac{4\pi}{5}}\sum_{m_1m_2}e_2^{m_1}e_2^{m_2}C^{2M}_{1m_11m_2}Y_{2M}(\hat{\mathbf{R}}),\\ &\sum_m C^{20}_{j-mj_1m}Y_{j-m}(\hat{\mathbf{k}})Y_{j_1m}(\hat{\mathbf{k}})=\sqrt{\frac{(2j+1)(2j_1+1)}{4\pi5}}C^{20}_{j0j_10}Y_{20}(\hat{\mathbf{k}}),\\ &\langle J_1'M_1'|Y_{2M}(\hat{\mathbf{R}})|J_1M_1\rangle=\int d\hat{\mathbf{R}}Y_{J_1M_1}Y_{2M}Y^*_{J_1'M_1'}=\sqrt{\frac{5(2J_1+1)}{4\pi(2J_1'+1)}}C^{J_1'0}_{J_1020}C^{J_1'M_1'}_{J_1M_12M}.\end{aligned} \tag{S36}$$

Using the Rayleigh expansion of a plane wave (eq. (25)) we get

$$\begin{aligned}\langle J_1M_1,\nu_1|e^{-\imath\alpha\mathbf{k}\cdot\mathbf{R}}|J_0M_0,0\rangle&=4\pi\sum_{jm}(-\imath)^jY^*_{jm}(\hat{\mathbf{k}})\langle J_1M_1|Y_{jm}(\hat{\mathbf{R}})|J_0M_0\rangle\langle\nu_1|j_j(\alpha kR)|0\rangle\\ &=4\pi\sum_{jm}(-\imath)^jY^*_{jm}(\hat{\mathbf{k}})\langle\nu_1|j_j(\alpha kR)|0\rangle\sqrt{\frac{(2J_0+1)(2j+1)}{4\pi(2J_1+1)}}C^{J_10}_{J_00j0}C^{J_1M_1}_{J_0M_0jm}.\end{aligned} \tag{S37}$$

[1] F. Gel'mukhanov, V. Carravetta, and H. Ågren, *Phys. Rev. B*, 1998, **58**, 2216.
[2] D. A. Varshalovich, A. N. Moskalev, and A. N. Khersonskii, *Quantum Theory of Angular Momentum*, World Scientific, Singapore, 1988.
[3] H. D. Cohen and U. Fano, *Phys. Rev.*, 1966, **150**, 30.
[4] F. Gel'mukhanov and H. Ågren, *Phys. Rev. A*, 1994, **49**, 4378.
[5] F. Gel'mukhanov, M. Odelius, S.P. Polyutov, A. Föhlisch, and V. Kimberg, *Rev. Mod. Phys.*, 2021, **93**, 035001.
[6] L.D. Landau and E.M. Lifshitz, *The Classical Theory of Fields*, Pergamon Press Oxford, England, 3d edn, 1971.
